# Antihypertensive treatment and risk of cancer: an individual participant data meta-analysis

**DOI:** 10.1016/S1470-2045(21)00033-4

**Published:** 2021-04

**Authors:** Emma Copland, Dexter Canoy, Milad Nazarzadeh, Zeinab Bidel, Rema Ramakrishnan, Mark Woodward, John Chalmers, Koon K Teo, Carl J Pepine, Barry R Davis, Sverre Kjeldsen, Johan Sundström, Kazem Rahimi, The Blood Pressure Lowering Treatment Trialists' Collaboration, A Adler, L Agodoa, A Algra, F W Asselbergs, N Beckett, E Berge, H Black, F P J Brouwers, M Brown, C J Bulpitt, B Byington, J Chalmers, W C Cushman, J Cutler, B R Davis, R B Devereaux, J Dwyer, R Estacio, R Fagard, K Fox, T Fukui, A K Gupta, R R Holman, Y Imai, M Ishii, S Julius, Y Kanno, S E Kjeldsen, J Kostis, K Kuramoto, J Lanke, E Lewis, J Lewis, M Lievre, L H Lindholm, S Lueders, S MacMahon, G Mancia, M Matsuzaki, M H Mehlum, S Nissen, H Ogawa, T Ogihara, T Ohkubo, C Palmer, A Patel, C J Pepine, M Pfeffer, N R Poulter, H Rakugi, G Reboldi, C Reid, G Remuzzi, P Ruggenenti, T Saruta, J Schrader, R Schrier, P Sever, P Sleight, J A Staessen, H Suzuki, L Thijs, K Ueshima, S Umemoto, W H van Gilst, P Verdecchia, K Wachtell, P Whelton, L Wing, M Woodward, Y Yui, S Yusuf, A Zanchetti, Z Y Zhang, C Anderson, C Baigent, BM Brenner, R Collins, D de Zeeuw, J Lubsen, E Malacco, B Neal, V Perkovic, B Pitt, A Rodgers, P Rothwell, G Salimi-Khorshidi, J Sundström, F Turnbull, G Viberti, J Wang

**Affiliations:** a Deep Medicine, Oxford Martin School, University of Oxford, Oxford, UK; b Nuffield Department of Women's and Reproductive Health, University of Oxford, Oxford, UK; c National Institute for Health Research Oxford Biomedical Research Centre, Oxford University Hospitals NHS Foundation Trust, Oxford, UK; d The George Institute for Global Health, University of New South Wales, Sydney, NSW, Australia; e Department of Epidemiology and Biostatistics, The George Institute for Global Health, Imperial College London, London, UK; f Department of Epidemiology, Johns Hopkins University, Baltimore, MD, USA; g Population Health Research Institute, Hamilton Health Sciences, McMaster University, Hamilton, ON, Canada; h College of Medicine, University of Florida, Gainesville, FL, USA; i School of Public Health, University of Texas, Houston, TX, USA; j Department of Cardiology, University of Oslo, Ullevaal Hospital, Oslo, Norway; k Department of Medical Sciences, Clinical Epidemiology, Uppsala University, Uppsala, Sweden

## Abstract

**Background:**

Some studies have suggested a link between antihypertensive medication and cancer, but the evidence is so far inconclusive. Thus, we aimed to investigate this association in a large individual patient data meta-analysis of randomised clinical trials.

**Methods:**

We searched PubMed, MEDLINE, The Cochrane Central Register of Controlled Trials, and ClinicalTrials.gov from Jan 1, 1966, to Sept 1, 2019, to identify potentially eligible randomised controlled trials. Eligible studies were randomised controlled trials comparing one blood pressure lowering drug class with a placebo, inactive control, or other blood pressure lowering drug. We also required that trials had at least 1000 participant years of follow-up in each treatment group. Trials without cancer event information were excluded. We requested individual participant data from the authors of eligible trials. We pooled individual participant-level data from eligible trials and assessed the effects of angiotensin-converting enzyme inhibitors (ACEIs), angiotensin II receptor blockers (ARBs), β blockers, calcium channel blockers, and thiazide diuretics on cancer risk in one-stage individual participant data and network meta-analyses. Cause-specific fixed-effects Cox regression models, stratified by trial, were used to calculate hazard ratios (HRs). The primary outcome was any cancer event, defined as the first occurrence of any cancer diagnosed after randomisation. This study is registered with PROSPERO (CRD42018099283).

**Findings:**

33 trials met the inclusion criteria, and included 260 447 participants with 15 012 cancer events. Median follow-up of included participants was 4·2 years (IQR 3·0–5·0). In the individual participant data meta-analysis comparing each drug class with all other comparators, no associations were identified between any antihypertensive drug class and risk of any cancer (HR 0·99 [95% CI 0·95–1·04] for ACEIs; 0·96 [0·92–1·01] for ARBs; 0·98 [0·89–1·07] for β blockers; 1·01 [0·95–1·07] for thiazides), with the exception of calcium channel blockers (1·06 [1·01–1·11]). In the network meta-analysis comparing drug classes against placebo, we found no excess cancer risk with any drug class (HR 1·00 [95% CI 0·93–1·09] for ACEIs; 0·99 [0·92–1·06] for ARBs; 0·99 [0·89–1·11] for β blockers; 1·04 [0·96–1·13] for calcium channel blockers; 1·00 [0·90–1·10] for thiazides).

**Interpretation:**

We found no consistent evidence that antihypertensive medication use had any effect on cancer risk. Although such findings are reassuring, evidence for some comparisons was insufficient to entirely rule out excess risk, in particular for calcium channel blockers.

**Funding:**

British Heart Foundation, National Institute for Health Research, Oxford Martin School.

## Introduction

Although evidence for the benefits of antihypertensive medication in the prevention of cardiovascular disease is well established,^1^ low adherence to treatment is a major barrier to effective blood pressure control.^2^ Non-compliance with antihypertensive medication is often due to concerns about possible adverse effects,^3^ including an increased risk of developing cancer.^4^, ^5^, ^6^, ^7^ Several pathways have been hypothesised to explain possible associations between raised blood pressure and cancer risk, but findings have been inconsistent and mainly based on observational studies.^7^, ^8^ Most concerns have been associated with off-target effects of specific drug classes, such as possible carcinogenic effects of angiotensin II receptor blockers (ARBs) on lung tissue and the photosensitising effect of thiazide diuretics that could increase the susceptibility of the skin to the effects of sunlight exposure.^9^, ^10^

A series of meta-analyses of randomised controlled trials, based on aggregate data, have investigated the association between class-specific antihypertensive treatment and risk of cancer, but findings have been conflicting. One study has suggested that using ARBs increases the risk of cancer,^4^ whereas two subsequent meta-analyses showed no such association.^11^, ^12^ Another meta-analysis of randomised controlled trials found no evidence linking any drug class with the incidence of any cancer,^12^ but an increased risk of cancer with the use of angiotensin-converting enzyme inhibitors (ACEIs) in combination with ARBs could not be ruled out. However, findings from existing meta-analyses based on summary statistics are limited by the study design, because such methods could not account for competing risks. Additionally, these analyses could not assess the timing of cancer events, since events occurring shortly after treatment initiation are unlikely to be causally linked to treatment since it is biologically plausible that a latency period exists between exposure to the medication and cancer occurrence.

**Research in context**

**Evidence before this study**

We searched PubMed, MEDLINE, The Cochrane Central Register of Controlled Trials, and ClinicalTrials.gov from Jan 1, 1966, to Sept 1, 2019, without language restrictions, for randomised controlled trials and meta-analyses investigating blood pressure lowering treatment. We searched MEDLINE using and expanding on the MeSH terms for “hypertension”, “blood pressure”, and “antihypertensive agents” including possible variations thereof and relevant antihypertensive drug classes. Our search identified 100 trials eligible for inclusion in the Blood Pressure Treatment Trialists' Collaboration. Of the trials and meta-analyses that reported cancer outcomes, no consistent associations were identified between any antihypertensive drug class and cancer risk.

**Added value of this study**

In this meta-analysis of individual patient-level data from 33 randomised controlled trials, to our knowledge, the one with the largest sample size to date, we found no compelling evidence that the use of any antihypertensive drug class had a significant effect on the risk of cancer when compared with placebo. Furthermore, we found no consistent evidence that the use of any antihypertensive drug class had a material effect on the risk of developing breast, colon, lung, prostate, or skin cancer. We found no association between risk estimates and longer durations of treatment (up to 4 years on average). The effect also did not vary across groups stratified by age, sex, body-mass index, smoking status, or previous antihypertensive use at baseline.

**Implications of all the available evidence**

Our study addresses a gap in the evidence for the safety of antihypertensive medication. Together with the established benefits of antihypertensive medication for the prevention of cardiovascular disease, our study provides evidence against antihypertensive treatment being associated with increased cancer risk. These findings are reassuring for patients and clinicians using these drugs and should encourage an improvement in adherence to antihypertensive medications. However, evidence for some cancer types was insufficient to entirely rule out the possibility of some excess risk, in particular, after a duration of treatment longer than that considered in our study.

The Blood Pressure Lowering Treatment Trialists' Collaboration (BPLTTC) is a collaboration of the principal investigators of major global clinical trials of pharmacological blood pressure lowering treatment, coordinated by the University of Oxford (Oxford, UK). The collaboration provides the most extensive individual patient-level dataset of blood pressure lowering trials currently available worldwide. Using the BPLTTC database, we aimed to investigate class-specific effects of antihypertensive drugs on the outcomes of cancer, cancer deaths, and site-specific cancers.

## Methods

### Study governance and data source

For this meta-analysis of individual participant-level data, we used the BPLTTC database,^13^, ^14^ which currently has access to individual participant data from randomised controlled trials identified as described in the search strategy and selection criteria section and the study protocol.^13^, ^14^ The study protocol was approved by the Steering Committee and Collaborators before the data was released for analysis and is available in the appendix (pp 29–39). Ethical approval for the current study was obtained from the Oxford Tropical Research Ethics Committee (OxTREC Reference 545–14).

### Search strategy and selection criteria

The search strategy and primary criteria for inclusion in the BPLTTC have been published previously^14^ and are reported in the appendix (pp 2–4). Briefly, we searched PubMed, MEDLINE, The Cochrane Central Register of Controlled Trials, and ClinicalTrials.gov for randomised controlled trials investigating pharmacological blood pressure lowering treatments published between Jan 1, 1966, and Sept 1, 2019. We searched MEDLINE using and expanding on the MeSH terms for “hypertension”, “blood pressure”, and “antihypertensive agents”, including possible variations thereof and relevant antihypertensive drug classes, without language restrictions. The full search strategy for MEDLINE is included in the appendix (p 10). Eligible trials for this study were randomised controlled trials comparing one blood pressure lowering drug class with a placebo, inactive control, or other blood pressure lowering drug. We also required that trials had at least 1000 participant years of follow-up in each treatment group and reported individual participant data on cancer events and timing of diagnosis during follow-up. We excluded trials that did not provide cancer event information.

### Data extraction

Two investigators (DC, MN) independently screened titles and abstracts for eligibility and any conflicts were resolved through discussion with a third investigator (KR). Individual participant data were requested from all eligible trials (full list of variables requested is reported in the appendix [pp 11–12]). Some trials included in the collaboration had previously reported numbers of cancer events, whereas others had not published this information previously. Analyses were confined to studies that compared one main drug class with a control group (or groups) and studies that compared more versus less intensive treatment regimens without a specific drug class group were excluded. All participants from eligible trials were included in the analysis. We used the Revised Cochrane risk-of-bias tool^15^ to assess the risk of bias of individual trials.

We extracted individual participant data for baseline characteristics (appendix pp 11–12) and follow-up blood pressure measurements, cancer events, and cancer deaths.

### Outcomes

The primary outcome was any cancer event, defined as the first occurrence of any cancer diagnosed after randomisation. Cancer events in the trials were reported using Classification of Diseases codes and Medical Dictionary for Regulatory Activities classifications. These cancer events include those prespecified as outcomes and those reported as adverse events in each trial. Secondary outcomes were deaths with cancer as the underlying cause and site-specific cancers. The site-specific cancers analysed included common cancers and subtypes that have previously been reported to be associated with blood pressure lowering treatment, comprising of breast, colorectal, lung, prostate, and skin cancers.^5^, ^6^, ^7^, ^8^, ^9^, ^10^, ^16^ We describe the source of these outcomes for each trial, and whether or not these outcomes have been adjudicated by an endpoint committee on the basis of certain criteria, in the appendix (pp 13–17).

### Data analysis

Characteristics of the participants included in each drug class comparison at baseline were described using summary statistics. All analyses were time-to-event analyses done using Cox proportional hazards models, stratified by trial, and were based on the intention-to-treat principle. In some trials, the exact dates of cancer diagnosis were not recorded in the trial database. In the absence of exact dates, the date of cancer diagnosis was approximated using the closest date to diagnosis on the basis of the date the cancer was first reported in the study or the date of death in participants for whom cancer had not been diagnosed or recorded before death with the underlying cause reported as cancer. Individuals were censored at date of death or last follow-up date. We used cause-specific fixed-effects Cox regression models for cancer events, with additional censoring for non-cancer deaths, to account for the competing risks. We fit cause-specific hazard models and Fine and Gray subdistribution hazard models to account for competing risk of non-cancer death. The primary analyses were done using data from cause-specific models, because they are considered more appropriate for assessing the causes of an event than Fine and Gray models.^17^ Proportional hazard assumptions were tested by plotting log-log plots and by assessment of Schoenfeld residuals.

We examined the effects of each antihypertensive drug class using the one-stage individual participant data meta-analysis framework.^18^, ^19^ In these prespecified analyses, the active group included participants who were randomly assigned to a specific antihypertensive drug class (ACEI, ARB, β blockers, calcium channel blockers, or thiazide diuretics) and the control group includes participants randomly assigned to all other comparator groups, including placebo, standard treatments, or other drug classes (or drug class combinations). Further details of treatment comparison groups are described in the appendix (pp 18–19). We estimated the heterogeneity of cancer risk effects across each of these comparisons using χ^2^ tests. We also did a network meta-analysis to investigate the class-specific effects of antihypertensives compared with a placebo reference group.^20^, ^21^, ^22^, ^23^ In this prespecified analysis, the effects of drug classes were analysed simultaneously by combining all available direct and indirect evidence across the network of studies.^20^, ^21^, ^22^, ^23^ Placebo-controlled trials contributed directly to the hazard ratio (HR) estimates of each antihypertensive drug class on cancer risk, and all other trials contributed indirectly. We reported the proportion of direct evidence in each comparison. We used fixed-effect network meta-analysis models, and assessed inconsistency across treatment effects using *Q* statistics. We have presented network graphs of all pairwise treatment comparisons in the network (appendix p 5). We have also reported the results for each pairwise comparison, because the network meta-analysis estimated the treatment effect of each drug class compared with each other drug class. Network meta-analyses were not done for site-specific outcomes due to small numbers of events from placebo-controlled trials.

To assess any temporal variation in risk, we did a post-hoc analysis to estimate the HR for each drug class according to specific timepoints during follow-up, and tested for heterogeneity and linear trend in risk across the follow-up duration. In the time-stratified analysis, patients contributed to the time of exposure at each time period until they developed the outcome or were censored. For cancer and cancer death outcomes, we prespecified subgroup analyses of the stratified effects of antihypertensive drug classes by baseline age, sex, smoking status, and body-mass index (BMI). We also stratified analyses based on previous use of antihypertensive medication at baseline, to test the hypothesis that true harmful effects are masked by widespread use of non-randomised treatment before trial participation. Heterogeneity of treatment effect across drug classes and subgroups were assessed using χ^2^ statistics. For the analyses stratified by follow-up period and patient characteristics, and analyses investigating site-specific cancer outcomes, we have presented unadjusted p values for heterogeneity and adjusted p values for multiple comparisons calculated using the Bonferroni method. We did the following sensitivity analyses: competing risk analysis using Fine and Gray subdistribution models to determine whether bias was introduced into the analysis due to competing risks; two-stage meta-analysis combining estimates from individual trials using the fixed-effect inverse-variance weighting approach to ensure that the HRs from the two-stage approach were comparable with those from the one-stage approach; and a comparison of the effects of each antihypertensive drug class on any cancer between trials that explicitly excluded cancer patients at baseline, and therefore only reported incident events, and those that did not exclude cancer patients at baseline and consequently might have reported recurrent events (appendix p 3).

We reported HRs with corresponding 95% CIs for all analyses, calculated from time-to-event models, and p values for all analyses of less than 0·05 were considered to indicate significance. All statistical analyses were done using R (version 3.3). This study is registered with PROSPERO (CRD42018099283).

### Role of the funding source

The funder of the study had no role in study design, data collection, data analysis, data interpretation, or writing of the report.

## Results

The systematic review identified 11 494 studies, from which 100 trials were considered potentially eligible for the BPLTTC studies. Individual patient data were obtained from 51 trials (appendix p 4). From these, 12 trials were excluded because data on cancer events during follow-up were unavailable (ie, only 39 reported on cancer outcomes). A further six trials were excluded because they did not include a drug class comparison group, therefore 33 trials^24^, ^25^, ^26^, ^27^, ^28^, ^29^, ^30^, ^31^, ^32^, ^33^, ^34^, ^35^, ^36^, ^37^, ^38^, ^39^, ^40^, ^41^, ^42^, ^43^, ^44^, ^45^, ^46^, ^47^, ^48^, ^49^, ^50^, ^51^, ^52^, ^53^, ^54^, ^55^, ^56^, ^57^, ^58^, ^59^, ^60^, ^61^, ^62^, ^63^, ^64^, ^65^, ^66^, ^67^, ^68^, ^69^, ^70^, ^71^, ^72^, ^73^, ^74^ including 260 447 individuals had cancer outcome data available and included a drug class comparison group, and thus met the inclusion criteria (table). Of the 33 trials included in the analysis, 16 (48%) trials that contributed to 11 833 (79%) of 15 012 cancer events had previously reported on cancer risk or had been included in aggregate meta-analyses of randomised controlled trials.^4^, ^11^, ^12^ 3251 (21%) cancer events from 12 trials included were published for the first time in this study. 11 trials explicitly excluded patients with cancer at baseline (2525 [17%] events; appendix pp 13–17). Cancer was a prespecified safety outcome in 13 trials that contributed 10 119 (67%) events (appendix p 13–17). In the remaining 20 trials (4965 [33%] events), cancer was identified routinely as part of adverse event reporting. In 13 trials (6663 [44%] of 15 012 events), an endpoint committee adjudicated cancer events (appendix pp 13–17). The risk of bias assessment indicated that 29 trials were at low risk of bias, and four trials had some risk of bias (appendix p 20).

**Table:** Characteristics of trials and participants

		Drug class comparison*					**All trials**
		ACEI *vs* other^24^, ^25^, ^26^, ^27^, ^28^, ^30^, ^31^, ^32^, ^35^, ^36^, ^37^, ^45^, ^46^, ^49^, ^50^, ^54^, ^55^, ^57^, ^62^, ^63^, ^64^, ^65^, ^70^	ARB *vs* other^29^, ^38^, ^39^, ^44^, ^53^, ^54^, ^58^, ^59^, ^60^, ^62^, ^63^, ^68^, ^69^, ^70^, ^72^, ^73^, ^74^	β blocker *vs* other^24^, ^25^, ^33^, ^34^, ^44^, ^47^, ^48^, ^58^, ^59^	Calcium channel blockers *vs* other^24^, ^25^, ^26^, ^27^, ^28^, ^30^, ^31^, ^33^, ^34^, ^35^, ^36^, ^37^, ^38^, ^39^, ^40^, ^41^, ^42^, ^43^, ^47^, ^48^, ^54^, ^56^, ^57^, ^60^, ^61^, ^66^, ^67^, ^70^, ^71^, ^73^, ^74^	Thiazide *vs* other^30^, ^31^, ^32^, ^40^, ^41^, ^44^, ^51^, ^52^, ^61^	
Trials		15	11	5	19	6	33
Participants		118 574	99 711	35 169	150 745	58 185	260 447
	Women	44 301 (37%)	37 941 (38%)	12 589 (36%)	69 399 (46%)	27 927 (48%)	106 453 (41%)
	Men	74 271 (63%)	61 769 (62%)	22 578 (64%)	81 344 (54%)	30 261 (52%)	154 489 (59%)
Participant age, years		66 (60–72)	67 (60–73)	64 (57–70)	66 (60–73)	68 (62–73)	66 (60–72)
Participant age at baseline, years							
	<65	50 864/118 569 (43%)	41 441/99 673 (42%)	19 152/35 169 (54%)	65 720/150 731 (44%)	20 108/58 185 (35%)	112 373/260 393 (43%)
	≥65	67 685/118 569 (57%)	58 232/99 673 (58%)	16 015/35 169 (46%)	85 009/150 731 (56%)	38 080/58 185 (65%)	148 517/260 393 (57%)
Ethnicity							
	White	70 174/104 648 (67%)	63 770/97 377 (65%)	29 154/34 073 (86%)	84 752/138 435 (61%)	25 962/55 781 (47%)	145 853/221 293 (66%)
	African American	15 799/104 648 (15%)	2746/97 377 (3%)	2096/34 073 (6%)	20 037/138 435 (14%)	13 686/55 781 (25%)	22 312/221 293 (10%)
	Hispanic	9684/104 648 (9%)	4091/97 377 (4%)	116/34 073 (<1%)	16 376/138 435 (12%)	6690/55 781 (12%)	21 000/221 293 (9%)
	Asian	9472/104 648 (9%)	23877/97 377 (25%)	3610/34 073 (11%)	17 096/138 435 (12%)	9443/55 781 (17%)	32 493/221 293 (15%)
	Other	613/104 648 (1%)	2873/97 377 (3%)	195/34 073 (1%)	755/138 435 (1%)	NA	3440/221 293 (2%)
Pre-treatment systolic blood pressure, mm Hg		147 (21)	149 (20)	166 (17)	155 (20)	151 (17)	151 (21)
Pre-treatment diastolic blood pressure, mm Hg		84 (11)	86 (12)	95 (10)	88 (11)	86 (10)	86 (11)
Trial duration, years		4·5 (4·0–5·1)	4·4 (3·1–4·9)	5·0 (4·5–5·8)	4·0 (2·8–5·2)	4·5 (3·7–5·5)	4·3 (3·0–5·0)
Previously on blood pressure lowering medication		78 018/93 064 (83%)	77 061/95 008 (81%)	25 546/34 073 (75%)	79 058/97 810 (81%)	46 265/54 054 (86%)	167 195/210 978 (79%)
Current smoker		19 519/118 413 (16%)	16 378/99 567 (16%)	9273/35 150 (26%)	30 739/150 463 (20%)	11 132/58 185 (19%)	47 199/260 269 (18%)
BMI, kg/m^2^		28 (5)	28 (5)	28 (5)	28 (5)	28 (6)	28 (5)
	<25	29 830/117 465 (25%)	31 800/99 340 (32%)	8949/35 033 (25%)	30 568/111 786 (27%)	15 871/57 435 (28%)	62 862/221 135 (28%)
	25–30	51 059/117 465 (43%)	41 924/99 340 (42%)	15 845/35 033 (45%)	46 248/111 786 (41%)	22 390/57 435 (39%)	95 361/221 135 (43%)
	≥30	37 040/117 465 (31%)	25 616/99 340 (26%)	10 237/35 033 (29%)	34 967/111 786 (31%)	19 172/57 435 (33%)	63 409/221 135 (29%)

Data are n, n (%), median (IQR), n/N (%), or mean (SD). ACEI=angiotensin-converting enzyme inhibitors. ARB=angiotensin II receptor blockers. NA=not available. BMI=body-mass index. The number of studies cited exceeds the total number of trials included in the mta-analysis because multiple references have been cited for some trials. Some percentages do not sum to 100 due to rounding.

* Drug class comparison groups are not mutually exclusive; some trials contribute data to more than one drug class (appendix pp 18–19).

15 trials (118 574 participants) included an ACEI drug class comparison; 11 trials (99 711 participants) included ARBs; five trials (35 169 participants) included β blockers; 19 trials (150 745 participants) included calcium channel blockers; and six trials (58 185 participants) included thiazides (table). The drug class comparisons were not mutually exclusive, since some trials contributed data to more than one comparison. For the network meta-analysis comparing drug classes against placebo, individual participant data for total cancer events was available for 72 812 participants from 13 placebo-controlled trials: seven included an ACEI treatment group, three included an ARB group, four included a calcium channel blocker group, and one included a thiazide diuretic group. Individual participant data for cancer death was available for 51 038 participants included in eight placebo-controlled trials: three included ACEIs, three included ARBs, and two included calcium channel blockers. No placebo-controlled trials were identified that included a β blockers comparison group. Eight trials included more than two treatment groups: six trials included three intervention groups and two trials included four treatment groups (appendix pp 13–19).

The median age of participants across all trials was 66 years (IQR 60–72). Additional participant characteristics stratified by drug class comparison are presented in the table. Details of participant characteristics for individual trials are included in the appendix (p 21).

**Figure 1:**
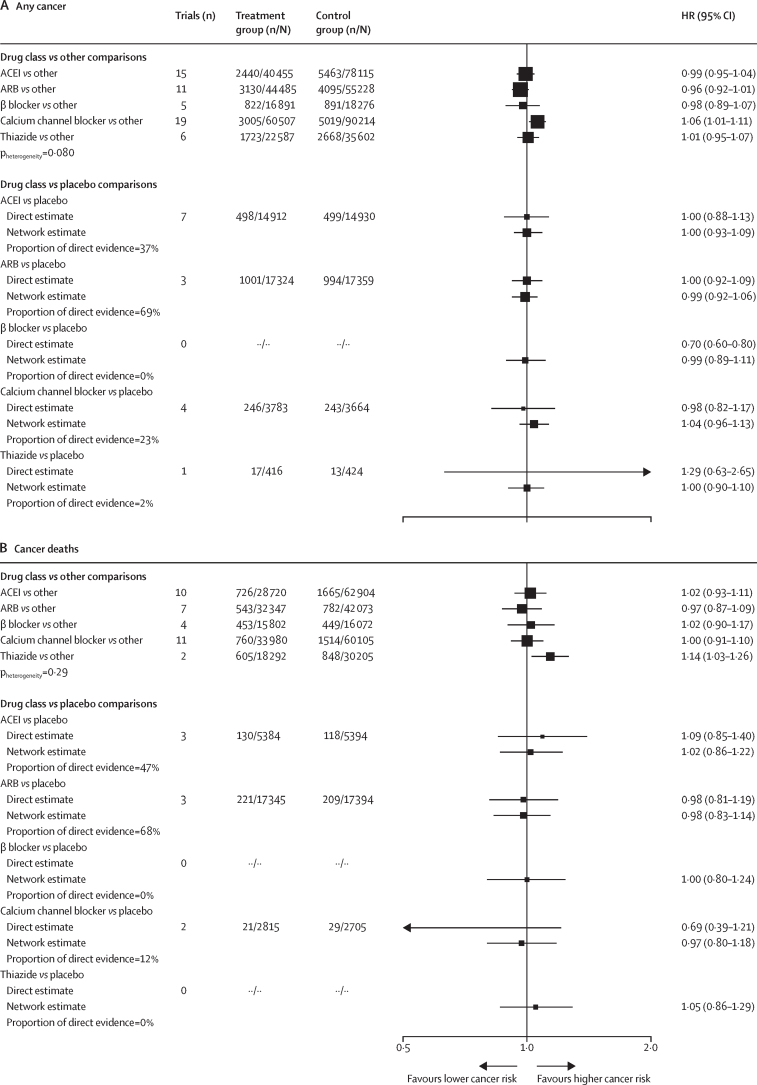
Effects of antihypertensive drug classes on risk of any cancer (A) and cancer death (B) Estimates based on individual participant-level data meta-analysis and network meta-analysis. n/N=number of events/number of participants**.** HR=hazard ratio. ACEI=angiotensin-converting enzyme inhibitors. ARB=angiotensin II receptor blockers. NA=not available.

After a median of 4·2 years (IQR 3·0–5·0) of follow-up, 15 012 participants were diagnosed with cancer across all 33 trials. We found no evidence of an association between antihypertensive drugs and any cancer when assessing all comparison groups (hazard ratio [HR] 0·99 [95% CI 0·95–1·04] for ACEIs; 0·96 [0·92–1·01] for ARBs; 0·98 [0·89–1·07] for β blockers; 1·01 [0·95–1·07] for thiazides), with the exception of calcium channel blockers (1·06 [1·01–1·11]; figure 1A). We also did not find an increased risk of cancer with use of any hypertensive drug in the network analysis using placebo as a comparator (HR 1·00 [95% CI 0·93–1·09] for ACEIs; 0·99 [0·92–1·06] for ARBs; 0·99 [0·89–1·11] for β blockers; 1·04 [0·96–1·13] for calcium channel blockers; 1·00 [0·90–1·10] for thiazides). In the one-stage meta-analysis, no evidence of effect modification by drug class was identified (p_heterogeneity_=0·080). In the network meta-analysis, no direct evidence of an effect was observed for any of the drug classes (figure 1A; appendix p 5).

In the one-stage meta-analyses comparing each drug class against all other comparators, no association was identified between antihypertensive treatments and cancer deaths, with the exception of thiazide diuretics, which were associated with an increased risk of death caused by cancer (figure 1B). In the network meta-analysis comparing each drug class against placebo, we found no associations between antihypertensive treatments and risk of cancer death. Across all associations, the network meta-analysis estimates were similar to the individual participant data meta-analysis estimates, with the exception of the effect of thiazide diuretics on the outcome of cancer death (figure 1B). Since no data were available on cancer death outcomes for any placebo-controlled trials with a thiazide diuretic drug class comparison, the network estimate was based entirely on indirect evidence from trials that included a thiazide diuretic group (two trials) or a placebo group, but not both.

**Figure 2:**
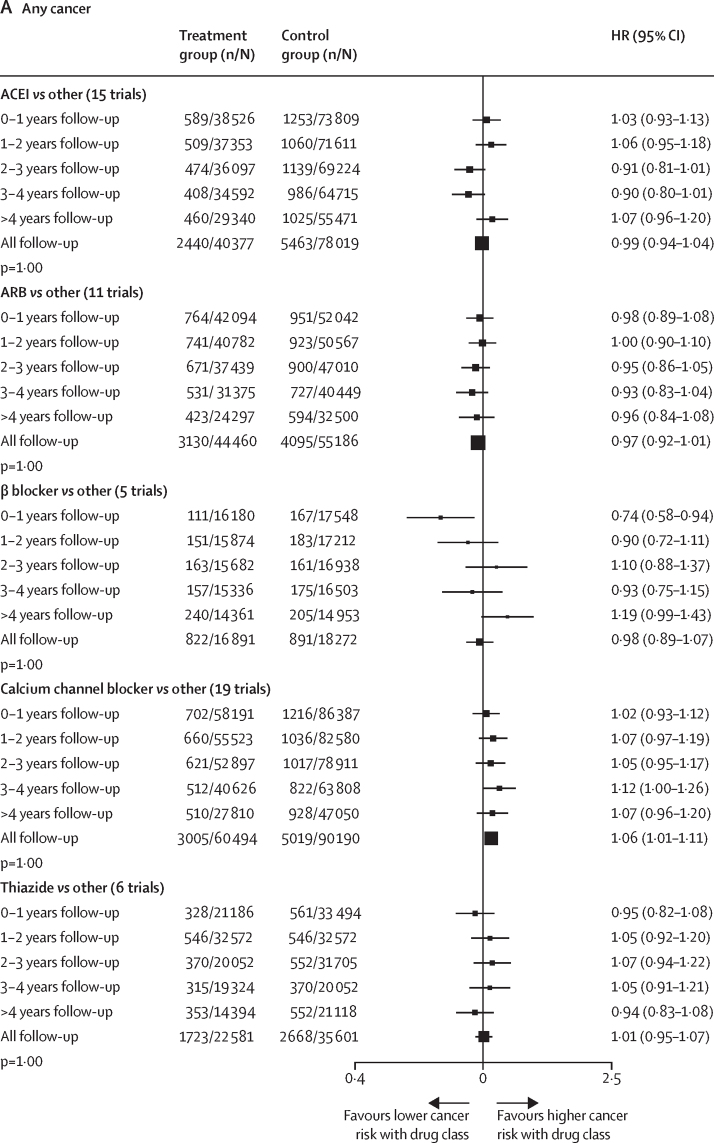

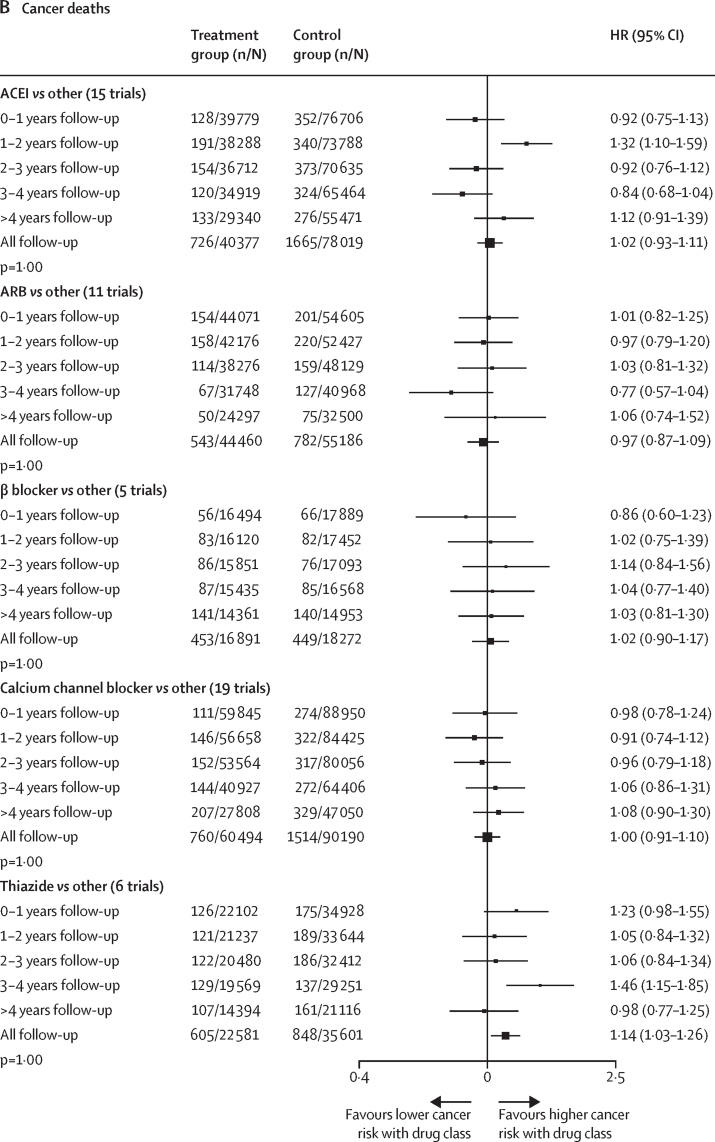
Effects of antihypertensive drug classes on risk of any cancer (A) and cancer death (B), stratified by follow-up duration p values are for linear trend and heterogeneity adjusted for multiple testing. n/N=number of events/number of participants. HR=hazard ratio. ACEI=angiotensin-converting enzyme inhibitors. ARB=angiotensin II receptor blockers.

In a post-hoc analysis, we also found no pattern of increasing or decreasing risk for any cancer or cancer death over time associated with any antihypertensive drug class (figure 2). Although there was some evidence of heterogeneity in treatment effect across different time periods for any cancer with ACEIs (p_heterogeneity_=0·004), calcium channel blockers (p_heterogeneity_=<0·0001), and thiazides (p_heterogeneity_=<0·0001), and for cancer death with calcium channel blockers (p_heterogeneity_=0·06) and thiazides (p_heterogeneity_=<0·001), there was no indication that the risk increased consistently over time (figure 2). In prespecified subgroup analyses, we found no evidence for variation in treatment effects across different age groups, sex, BMI categories, smoking status, or previous use of antihypertensive drugs (all p_heterogeneity_>0·10; appendix pp 6–8). The direct and network estimates from all pairwise comparisons of individual drug classes and placebo are presented in the appendix (p 22). We found no evidence for inconsistency in treatment effects across the network for any cancer or cancer death outcomes (p=0·60 for any cancer; p=0·88 for cancer death).

**Figure 3:**
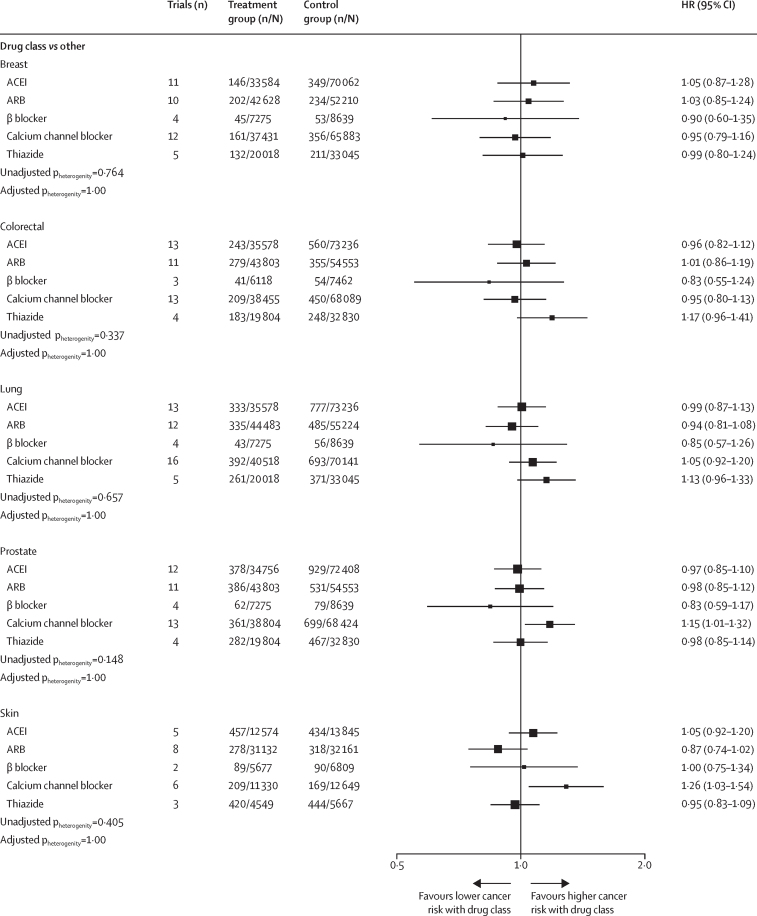
Effects of antihypertensive drug classes on risk of site-specific cancers Unadjusted p values for heterogeneity and p values adjusted for multiple comparisons are presented. n/N=number of events/number of participants. HR=hazard ratio. ACEI=angiotensin-converting enzyme inhibitors. ARB=angiotensin II receptor blockers.

We examined the effects of antihypertensive drug classes on risks of breast, colorectal, lung, prostate, and skin cancer compared with all other comparators (figure 3). Across all drug classes and site-specific cancers, we found no evidence of any associations, with the exception of calcium channel blockers which were associated with increased risk of prostate and skin cancers. The excess risks for calcium channel blockers on prostate and skin cancers were driven by the comparison of calcium channel blockers compared with ARBs (data not shown). We also examined these effects according to duration of follow-up and found no consistent temporal pattern in the risks for all drug classes (all p=1·00; data not shown).

In the two-stage meta-analysis, the HRs were comparable in magnitude with the results of the one-stage meta-analysis (appendix p 9). We also found that the subdistribution HRs from the Fine and Gray models were comparable to the cause-specific HRs, thus there was no sign of bias due to competing risks (data not shown). In the sensitivity analysis comparing the effects of antihypertensive drug classes on any cancer between trials that explicitly excluded cancer patients at baseline and those that did not, no significant heterogeneity in treatment effects was identified for any drug class compared with all other comparators (p_heterogeneity_=0·99 for ACEIs; p_heterogeneity_=0·78 for ARBs; p_heterogeneity_=0·55 for β blockers; p_heterogeneity_=0·40 for calcium channel blockers; p_heterogeneity_=0·17 for thiazides; appendix p 23).

## Discussion

In this study, we found no consistent evidence that the use of antihypertensive medication overall increased the risk of any cancer or cancer death. We also found no strong evidence that the use of any particular antihypertensive drug class had a consistent effect on the risk of developing breast, colon, lung, prostate, or skin cancer. These findings were further corroborated in the network meta-analyses based on the direct and indirect comparisons of drug classes with placebo, and in the time-stratified analyses, which showed no evidence of increasing or decreasing effects over time. However, the excess risks identified for calcium channel blockers on any cancer, prostate cancer, and skin cancer and for thiazide diuretics on cancer death in some analyses requires further investigation in clinical trials with a larger number of events, particularly for placebo-controlled comparisons.

Although several observational studies have previously reported an association between cancer risk and increased blood pressure or its treatment,^5^, ^6^, ^7^, ^16^, ^75^, ^76^ evidence based on randomised data is scarce, and meta-analyses of randomised evidence are mainly based on analysis of published summary statistics.^4^, ^11^, ^12^ Such study designs cannot account for competing risks, or investigate cancer events across different durations of follow-up. Little evidence is available from meta-analyses of published findings from randomised controlled trials on the effects on site-specific cancers because it is unlikely that a single trial would have sufficient statistical power to report these effects. Due to the large number of trials included in the BPLTTC database with individual participant data available, our study also addresses the paucity of evidence on antihypertensive drug use and cancer risk among important patient subgroups, and found no significant variation in the effects on any cancer across groups defined by age, sex, BMI, smoking status, or previous antihypertensive use with any antihypertensive drug class, indicating that any cancer-related adverse effects were unlikely to have been masked by widespread use of non-randomised treatment before trial participation.

Several hypotheses have been posited linking the pathways of specific drug classes to cancer, independently of changes in blood pressure.^9^, ^10^ There has been a concern around the potential association between thiazide diuretics and skin cancer risk due to the photosensitising properties of thiazides and harmful effects identified in several observational studies;^7^ however, our findings do not support an association between the use of thiazides and skin cancers. Other studies have also suggested that blockade of the renin–angiotensin system by ACEIs and ARBs might have a protective effect against a broad range of cancer types,^77^ including lung, breast, and prostate cancers,^78^ by affecting cell proliferation, angiogenesis, and apoptosis.^79^ However, we found no significant associations between any of these drug classes and risk of any cancers. Our findings suggesting a potential increased risk of any, prostate, or skin cancers with use of calcium channel blockers and cancer death with thiazides were unexpected considering that no compelling evidence exists with regard to plausible mechanisms that would affect carcinogenesis in these parts of the body with use of these drugs.^75^, ^80^ However, our detailed analyses and the absence of plausible mechanisms suggest that calcium channel blockers or thiazides are unlikely to cause such cancers. Comparison of a single drug class against all other groups is limited by uncertainty regarding whether the apparent excess risk is a true effect of the intervention or a reflection of a potentially beneficial effect of the drug class in the comparison group (which by chance will differ for different classes). In the case of thiazide diuretics, a larger number of trials providing cancer death data is required to investigate this association further, since only two trials contributed data to this analysis. In the case of calcium channel blockers, the excess risks identified were primarily driven by the comparison of calcium channel blockers against ARBs, which in turn seems to have been driven by data from a single trial (VALUE^73^, ^74^). Although no significant heterogeneity was identified across trials with a calcium channel blocker comparison in two-stage meta-analysis, the VALUE trial (calcium channel blocker *vs* ARB comparison), was an important driver of the excess risk for calcium channel blockers compared with all other comparators in the main analysis. To address this issue, we compared individual drug classes with placebo. Because of the relatively small number of placebo-controlled trials available for most drug classes, we did individual participant data network meta-analyses to estimate these effects. The results showed no evidence of any effects of drug classes on cancer risk when compared with placebo. This finding, together with the time-stratified analyses results, and the absence of heterogeneity in treatment effects across drug classes provide evidence against any class-specific effects on the risk of developing cancer. Consequently, it is possible that any variation around the null could be due to chance. However, these detailed and robust analyses have inadequate power to detect a statistical difference, particularly for site-specific cancers.

A key strength of this study was the use of individual participant data from the largest dataset of randomised controlled trials of antihypertensive drug treatments available to date, to our knowledge. Previously, a large meta-analysis of randomised controlled trials investigated the risk of cancer associated with antihypertensive treatment, but it was based on aggregate data^12^ and one study that analysed individual participant-level data only included 28 787 participants with 1823 cancer events.^4^ The number of participants included in our meta-analysis was nearly ten times higher and the number of cancer events was more than 13 000 higher than that included in the previous meta-analysis based on individual participant-level data, enabling a more detailed analysis to be done than previously possible. Another important strength of this study was that we had access to unpublished cancer event data collected during follow-up, and additional information from most trials on cancer subtypes, date of diagnosis, and information on multiple diagnoses in individual participants. Since we had access to time-to-event data, we were able to assess any trend in cancer risk over time, an analysis that has not been possible previously using randomised data. This analysis allowed us to account for the latency period between exposure to the antihypertensive drug and occurrence of cancer, since events diagnosed early during follow-up are less likely to be linked to the study medication. The results of this analysis suggested that there was no increased risk of cancer with continued treatment during the follow-up period. Thus, our study provides the most compelling evidence to date for the safety of antihypertensive drugs with respect to cancer and cancer subtypes that we have considered.

A limitation of this study was that we did not have access to individual participant data for all trials that were eligible for inclusion in the BPLTTC database. Therefore, although we had access to a larger number of cancer events from randomly assigned participants than did previous studies, some analyses involving cancer mortality or site-specific cancer outcomes were based on relatively small numbers of events, resulting in greater uncertainty around the risk estimates. For the same reason, our pre-defined protocol excluded analyses of uncommon cancer sites. The source of cancer outcomes varied across trials. Some trials reported prespecified cancer outcomes whereas others captured cancer events through routine adverse event reporting, and less than half of the trials adjudicated cancer events. However, previous evidence^81^, ^82^ has suggested that adjudication of common outcomes does not have an impact on relative treatment effects because any misclassification is expected to be consistent across treatment groups. Because of the paucity of data on baseline cancer history, we were unable to determine whether all cancer outcomes were incident events. However, our sensitivity analysis, stratified by explicit exclusion of cancer patients at baseline, suggested that there were no differences in the relative treatment effects in trials that excluded cancer patients compared with those that did not. Investigators across many trials were also allowed to prescribe additional non-study antihypertensive treatments to participants whose blood pressure had not been controlled sufficiently with the study drug. In cases where the treatment and control groups were systemically prescribed different classes of drugs (either by design or chance), this could lead to the underestimation of each drug class effect on the outcomes. Another potential limitation was that class-specific categorisation of antihypertensive medication might have diluted the effects of individual drugs that act via different biological pathways. Additionally, our study was based on a median follow-up duration of 4 years, which might not be sufficient for some cancers to develop. Hence, it would be prudent for future trials to continue collecting outcomes, including cancer, long after the trial has ended to allow the investigation of off-target effects of antihypertensive drugs. In our analyses stratified by follow-up duration, we found no evidence of an increasing risk with more years of exposure to the treatment; however, studies with longer durations might be necessary to rule out any association with long-term antihypertensive use.

Our study has addressed an ongoing controversy about the safety of blood pressure lowering medication with respect to cancer risk, using the largest sample of individual-level randomised evidence on blood pressure lowering treatment to date, to our knowledge. In our detailed analyses, we found no evidence that the use of antihypertensive medication has any substantial effect on cancer risk, although we could not rule out potential class-specific effects for calcium channel blockers and thiazide diuretics. This finding was consistent across patients with a wide range of baseline characteristics, therefore our study addresses a gap in the evidence for the safety of antihypertensive medication. It is estimated that between 30% and 50% of individuals have poor adherence to these drugs, partly because of concerns around the harmful effects that long-term use of antihypertensive medications might cause.^2^, ^3^ The main implication of our study is that patients using antihypertensive medication should continue to take their medications because concerns about increased cancer risk seem to be unfounded.

## Data sharing

The BPLTTC is governed by the University of Oxford's policies on research integrity and codes of practice and follows the university's policy on the management of research data and records. Scientific activities based on the BPLTTC dataset are overseen by the BPLTT Steering Committee. All data shared with the BPLTTC will be considered confidential and will not be provided to any third party. Requests for data should be made directly to the data custodians of individual trials.
