# Supplementary material for: Antihypertensive treatment and risk of cancer: an individual participant data meta-analysis
Source: Lancet Oncol. 2021 Apr;22(4):558–70. doi: 10.1016/S1470-2045(21)00033-4 (PMC8024901; doi:10.1016/S1470-2045(21)00033-4)
Supplement: Supplementary appendix [file mmc1.pdf]

THE LANCET
Oncology

Supplementary appendix

This appendix formed part of the original submission and has been peer reviewed. We post it as supplied by the authors.

Supplement to: Copland E, Canoy D, Nazarzadeh M, et al. Antihypertensive treatment and risk of cancer: an individual participant data meta-analysis. *Lancet Oncol* 2021; **22:** 570–82.

Web-only supplement

Table of contents

Working group…………...………………………………………………………………………….p 2

Supplementary methods……………………………………………………………………………….p 2

Supplementary figures

Supplementary Figure 1. PRISMA diagram for included trials.………………………………………...p 4

Supplementary Figure 2. Network of treatment comparisons ………………………………………...p 5

Supplementary Figure 3. Effects of antihypertensive drugs on any cancer compared against all other comparators and placebo, stratified by baseline characteristics of participants………………………………….…p 6

Supplementary Figure 4. Two-stage individual participant-level meta-analysis…….……….……….……p 9

Supplementary tables

Supplementary Table 1. MEDLINE search strategy for BPLTTC……………………………………...p 10

Supplementary Table 2. Full list of variables requested from participating trials…………………...………p 11

Supplementary Table 3. Characteristics of individual trials included in study. ………………………………p 13

Supplementary Table 4. List of trials and interventions ………………………………………………..p 18

Supplementary Table 5. Risk of bias assessment for individual trials…………………………………p 20

Supplementary Table 6. Characteristics of participants at baseline for each trial.…………………….…….…p 21

Supplementary Table 7. Effects of antihypertensive drug classes on the risk of any cancer and cancer death, based on direct comparison and network meta-analysis estimates.………………………………………p 22

Supplementary Table 8. Effects of antihypertensive drug classes on any cancer stratified by explicit exclusion of cancer patients at baseline.…………………………..……………………………...…………..p 23

List of collaborating trialists……………………………………………………………………p 24

References……………………………………………………………………………………..p 26

BPLTTC research protocol...…………………………………………………………………p 29

Working group

Emma Copland, MSc,[1-3] Dexter Canoy, MD,[1-3] Milad Nazarzadeh, MSc,[1,2] Zeinab Bidel, MSc,[1-3] Rema Ramakrishnan, PhD,[1,2] Prof Mark Woodward, PhD,[4-6] Prof John Chalmers, MD,[4] Prof Koon K. Teo, MD,[7] Prof Carl J. Pepine, MD,[8] Prof Barry R. Davis, MD,[9] Prof Sverre Kjeldsen, MD[10], Prof Johan Sundström, MD[11], Prof Kazem Rahimi, FRCP[1-3]

[1] Deep Medicine, Oxford Martin School, University of Oxford, Oxford, UK
[2] Nuffield Department of Women's and Reproductive Health, University of Oxford, Oxford, UK
[3] NIHR Oxford Biomedical Research Centre, Oxford University Hospitals NHS Foundation Trust, Oxford, UK
[4] The George Institute for Global Health, University of New South Wales, Sydney, Australia
[5] The George Institute for Global Health, Department of Epidemiology and Biostatistics, Imperial College, London, UK
[6] Department of Epidemiology, Johns Hopkins University, Baltimore, Maryland, USA
[7] Population Health Research Institute, Hamilton Health Sciences, McMaster University, Hamilton, Ontario, Canada
[8] College of Medicine, University of Florida, Gainesville, Florida, USA
[9] The University of Texas School of Public Health, Houston, Texas, USA
[10] Department of Cardiology, University of Oslo, Ullevaal Hospital, Oslo, Norway
[11] Department of Medical Sciences, Clinical Epidemiology, Uppsala University, Uppsala, Sweden

Supplementary methods

Eligibility criteria

Trials were eligible for inclusion in the Blood Pressure Lowering Trialists' Collaboration (BPLTTC) if one of the following criteria were met:

- Randomization of patients between a blood pressure-lowering agent and a placebo arm, or other inactive control
- Randomization of patients between various blood pressure-lowering intensities
- Randomization of patients between various antihypertensive drugs

A minimum of 1,000 participant years of follow-up was required in each randomly allocated arm for a trial to be eligible. There was no restriction on the publication date, setting or drug that was investigated.

The following types of trial were excluded:

- Trials exclusively conducted in patients with heart failure or short-term interventions in patients with acute myocardial infarction or other acute settings
- Trials with non-pharmacological interventions of blood pressure-lowering without a drug comparison arm
- Trials without a clearly defined randomization process
- Trials that did not provide cancer event information were further excluded from this analysis

Identifying studies

Potential eligible trials were identified through a systematic review. The search was restricted to randomised controlled trials or meta-analyses. There were no language restrictions. PubMed/MEDLINE, The Cochrane Central Register of Controlled Trials and ClinicalTrials.gov were searched covering the periods between 1 January 1966 and 1 June 2018. The time period was extended to 1 September 2019 for the current analysis. Reference lists of eligible studies, related meta-analyses and clinical trial registries were hand-searched to identify further studies. This systematic review protocol was registered with PROSPERO[1] (CRD42018099283). The search strategy for MEDLINE is presented in Supplementary Table 1 (p 10). The overall search strategy underlying the BPLTTC is summarized in Supplementary Figure 1 (p 4). 100 eligible trials were identified.

Study selection

Two investigators conducted independent searches and screened the publication titles and abstracts to assess their eligibility (DC, MN). Full manuscripts of potential eligible studies were requested and assessed based on the eligibility criteria. Disagreements were resolved through discussion with a third investigator (KR).

Data collection, transfer and storage

Individual participant-level data (IPD) was requested from investigators of newly identified trials as well as existing BPLTT collaborators. The full list of variables that were sought is presented in Supplementary Table 2 (p 11). As of December 2020, the collaboration had acquired data from 51 trials comprising 352,744 participants. Data are still being sought for the remaining 49 trials, however, many of these trials were published many years ago and the identification of a data guardian or electronic trial database has proven challenging.

Trial data were transferred using a secure file transfer system and stored on a secure server at the University of Oxford, to comply with data protection regulations and data sharing agreements. Access to the data is restricted to investigators directly involved in the research and can only be used for the exclusive purpose of the study that has been pre-approved by the BPLTTC Steering Committee.

Data cleaning and harmonization

The process of data cleaning and harmonization involved creating a database with all information obtained from investigators using the data dictionaries provided. Individual trial data, including number of participants, baseline characteristic variables, follow-up and outcome data, were checked and verified by comparing against published data. Prepared data were also checked for consistency and completeness. Investigators were contacted if any inconsistencies or missing data was identified.

Sensitivity analysis

In our sensitivity analyses, we repeated the primary analysis using Fine-Gray models and compared the subdistribution hazard ratios from these models to the cause-specific hazard ratios to determine whether bias was introduced into the analysis due to the competing risk of non-cancer death. Fine and Gray subdistribution models were not selected as the models for the primary analysis as they are less appropriate for addressing aetiological questions, such as the association between an exposure and the risk of an outcome, than cause-specific hazard models.[2] We also conducted a two-stage meta-analysis and compared the results against those from the one-stage meta-analysis investigating the effect of each antihypertensive drug class on cancer risk. In the two-stage meta-analysis, the estimates for each trial were combined using the fixed-effect inverse-variance weighting approach. We also compared the class-specific effects of antihypertensive drug classes on any cancer between trials that explicitly excluded cancer patients at baseline and those that did not.

Supplementary figures

Supplementary Figure 1. PRISMA diagram for included trials.

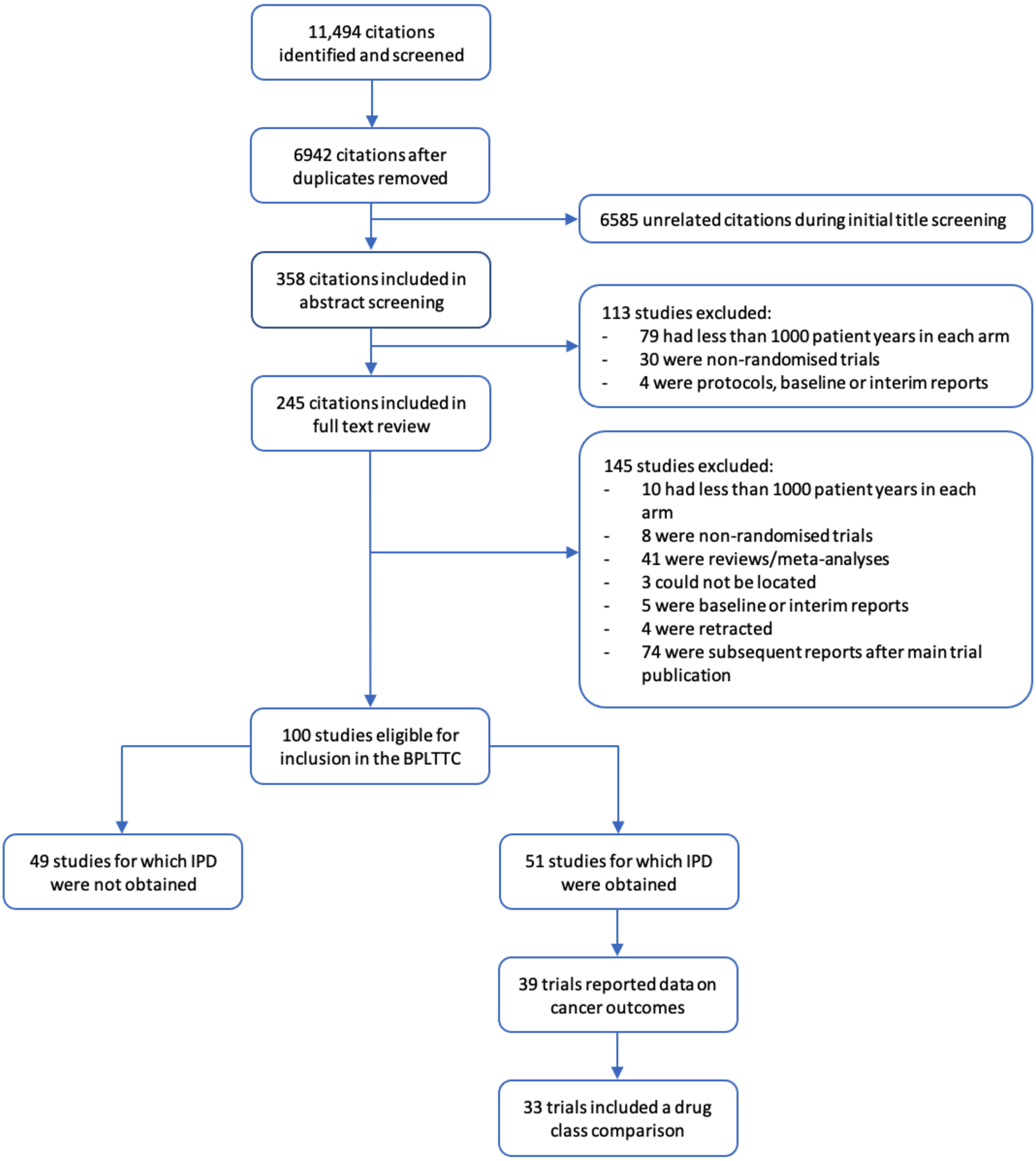

BPLTTC=Blood Pressure Lowering Treatment Trialists' Collaboration. IPD=individual participant-level data.

Supplementary Figure 2. Network of treatment comparisons for a) any cancer and b) cancer death. The number associated with each line represents the number of trials providing a direct comparison between the drug classes connected by the line.

A) Any cancer

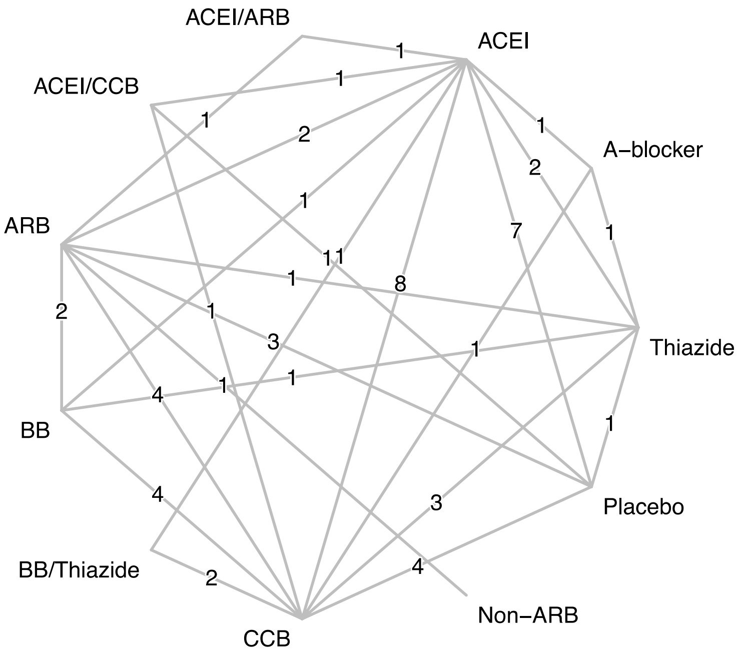

B) Cancer death

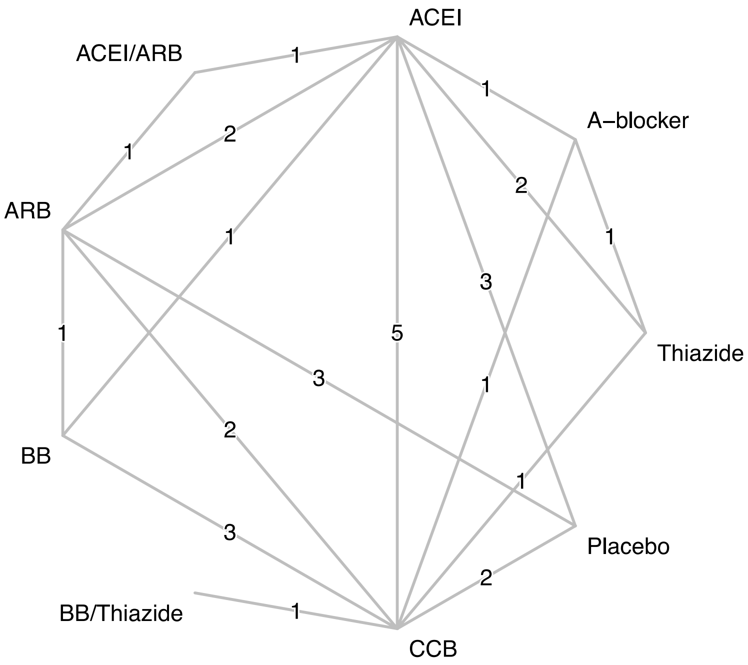

A-blocker=α-blocker. ACEI=angiotensin-converting enzyme inhibitors. ARB=angiotensin-II receptor blockers. BB=ß-blockers. CCB=calcium channel blockers.

Supplementary Figure 3. Class-specific effects of antihypertensive drugs on any cancer compared against all other comparators and placebo, stratified by baseline characteristics of participants.
Adjusted P values for heterogeneity indicate adjustment for multiple comparisons.

A. ACEI vs all other comparators

By baseline characteristic	N trials	Number of events / participants: Treatment	Number of events / participants: Control	HR (95% CI)
**Age**				
Less than 65	13	701 / 17503	1613 / 33361	0.98 (0.89, 1.07)
65 and over	15	1749 / 22950	3862 / 44755	1.00 (0.94, 1.06)
			Heterogeneity:	*unadjusted P=0.263, adjusted P=1*
**Gender**				
Male	15	1756 / 26415	3862 / 47856	0.98 (0.92, 1.03)
Female	15	694 / 14038	1613 / 30263	1.03 (0.94, 1.13)
			Heterogeneity:	*unadjusted P=0.426, adjusted P=1*
**Body mass index (kg/m^2)**				
Less than 25	15	641 / 10532	1318 / 19257	1.05 (0.96, 1.16)
25−30	14	1094 / 17899	2364 / 33007	0.98 (0.91, 1.06)
30 and over	14	704 / 11668	1761 / 25102	0.96 (0.88, 1.05)
			Heterogeneity:	*unadjusted P=0.713, adjusted P=1*
**Smoking status**				
Current smoker	15	497 / 6455	1261 / 13643	0.96 (0.87, 1.07)
Ex or never smoker	15	1953 / 33954	4213 / 64361	1.00 (0.95, 1.06)
			Heterogeneity:	*unadjusted P=0.154, adjusted P=1*
**Antihypertensive drug use**				
Yes	13	1962 / 29247	4599 / 59971	0.99 (0.94, 1.05)
No	14	392 / 9001	665 / 13739	1.00 (0.89, 1.12)
			Heterogeneity:	*unadjusted P=0.263, adjusted P=1*

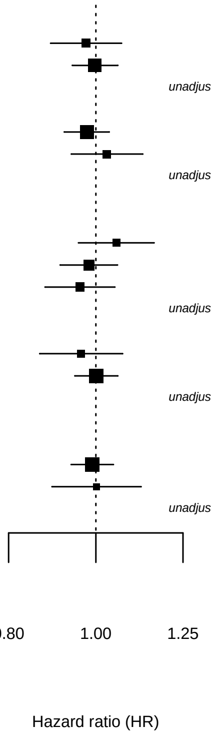

B. ARB vs all other comparators

By baseline characteristic	N trials	Number of events / participants: Treatment	Number of events / participants: Control	HR (95% CI)
**Age**				
Less than 65	11	848 / 18284	1138 / 23157	0.97 (0.89, 1.06)
65 and over	11	2306 / 26189	2994 / 32043	0.96 (0.91, 1.02)
			Heterogeneity:	*unadjusted P=0.908, adjusted P=1*
**Gender**				
Male	11	2174 / 27231	2912 / 34538	0.97 (0.92, 1.03)
Female	11	980 / 17253	1223 / 20688	0.94 (0.87, 1.03)
			Heterogeneity:	*unadjusted P=0.911, adjusted P=1*
**Body mass index (kg/m^2)**				
Less than 25	11	867 / 14036	1153 / 17764	0.96 (0.88, 1.05)
25−30	11	1414 / 18796	1800 / 23128	0.99 (0.92, 1.06)
30 and over	11	866 / 11506	1168 / 14110	0.93 (0.85, 1.02)
			Heterogeneity:	*unadjusted P=0.593, adjusted P=1*
**Smoking status**				
Current smoker	11	573 / 7648	727 / 9307	1.02 (0.92, 1.14)
Ex or never smoker	11	2581 / 36781	3408 / 45830	0.95 (0.90, 1.00)
			Heterogeneity:	*unadjusted P=0.665, adjusted P=1*
**Antihypertensive drug use**				
Yes	9	2646 / 34052	3589 / 43009	0.95 (0.90, 1.00)
No	10	459 / 8078	500 / 9868	1.05 (0.92, 1.19)
			Heterogeneity:	*unadjusted P=0.380, adjusted P=1*

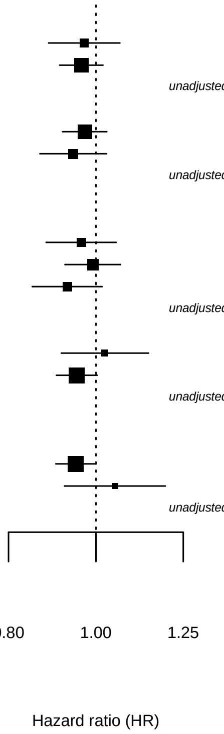

C. BBs vs all other comparators

By baseline characteristic	N trials	Number of events / participants: Treatment	Control	HR (95% CI)
**Age**				
Less than 65	6	260 / 9108	280 / 10044	1.01 (0.85, 1.20)
65 and over	6	562 / 7783	615 / 8232	0.95 (0.85, 1.07)
			Heterogeneity:	*unadjusted P=0.541, adjusted P=1*
**Gender**				
Male	6	515 / 10931	566 / 11647	0.97 (0.86, 1.09)
Female	6	307 / 5960	329 / 6629	0.99 (0.85, 1.16)
			Heterogeneity:	*unadjusted P=0.683, adjusted P=1*
**Body mass index (kg/m^2)**				
Less than 25	6	226 / 4099	265 / 4850	0.99 (0.83, 1.18)
25−30	6	351 / 7706	397 / 8139	0.90 (0.78, 1.04)
30 and over	6	236 / 5012	228 / 5225	1.07 (0.89, 1.28)
			Heterogeneity:	*unadjusted P=0.346, adjusted P=1*
**Smoking status**				
Current smoker	6	216 / 4461	222 / 4812	1.02 (0.85, 1.23)
Ex or never smoker	6	606 / 12419	673 / 13456	0.96 (0.86, 1.07)
			Heterogeneity:	*unadjusted P=0.598, adjusted P=1*
**Antihypertensive drug use**				
Yes	6	615 / 12730	659 / 13879	1.00 (0.90, 1.12)
No	5	207 / 4161	236 / 4397	0.90 (0.75, 1.09)
			Heterogeneity:	*unadjusted P=0.344, adjusted P=1*

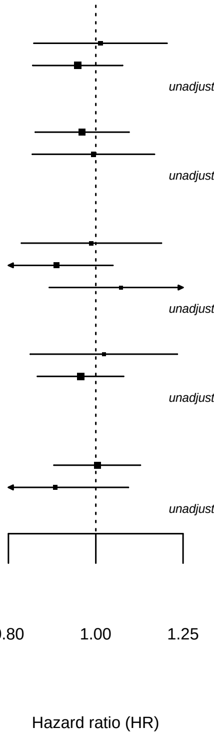

D. CCB vs all other comparators

By baseline characteristic	N trials	Number of events / participants: Treatment	Control	HR (95% CI)
**Age**				
Less than 65	17	819 / 26623	1428 / 39097	1.05 (0.96, 1.15)
65 and over	19	2189 / 33887	3592 / 51122	1.06 (1.01, 1.13)
			Heterogeneity:	*unadjusted P=0.269, adjusted P=1*
**Gender**				
Male	19	1897 / 32926	3165 / 48418	1.08 (1.02, 1.15)
Female	19	1111 / 27594	1855 / 41805	1.03 (0.95, 1.11)
			Heterogeneity:	*unadjusted P=0.990, adjusted P=1*
**Body mass index (kg/m^2)**				
Less than 25	17	674 / 11992	1129 / 18576	1.08 (0.98, 1.19)
25−30	16	1039 / 17305	1896 / 28943	1.04 (0.96, 1.12)
30 and over	16	788 / 12027	1494 / 22941	1.07 (0.98, 1.18)
			Heterogeneity:	*unadjusted P=0.700, adjusted P=1*
**Smoking status**				
Current smoker	19	755 / 12739	1290 / 18577	1.07 (0.98, 1.18)
Ex or never smoker	19	2249 / 47660	3729 / 71485	1.05 (1.00, 1.11)
			Heterogeneity:	*unadjusted P=0.260, adjusted P=1*
**Antihypertensive drug use**				
Yes	12	1995 / 28697	3748 / 51275	1.05 (0.99, 1.11)
No	12	317 / 7483	470 / 10969	1.12 (0.96, 1.30)
			Heterogeneity:	*unadjusted P=0.947, adjusted P=1*

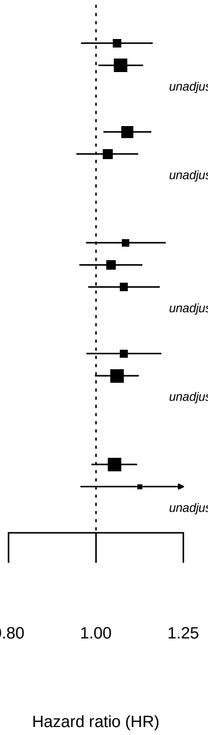

E. Thiazide diuretics vs all other comparators

By baseline characteristic	N trials	Number of events / participants: Treatment	Control	HR (95% CI)
**Age**				
Less than 65	4	464 / 7194	707 / 12914	1.09 (0.97, 1.22)
65 and over	6	1263 / 15391	1963 / 22689	0.98 (0.91, 1.05)
			Heterogeneity:	*unadjusted P=0.095, adjusted P=0.963*
**Gender**				
Male	6	1116 / 11615	1718 / 18646	1.03 (0.95, 1.11)
Female	6	611 / 10970	952 / 16957	0.98 (0.88, 1.08)
			Heterogeneity:	*unadjusted P=0.466, adjusted P=1*
**Body mass index (kg/m^2)**				
Less than 25	6	442 / 6558	699 / 9315	0.92 (0.81, 1.03)
25−30	6	687 / 8667	1081 / 13723	1.00 (0.91, 1.10)
30 and over	6	585 / 7089	866 / 12083	1.09 (0.98, 1.21)
			Heterogeneity:	*unadjusted P=0.177, adjusted P=1*
**Smoking status**				
Current smoker	6	433 / 4152	704 / 6978	0.99 (0.88, 1.11)
Ex or never smoker	6	1294 / 18433	1966 / 28625	1.01 (0.94, 1.09)
			Heterogeneity:	*unadjusted P=0.773, adjusted P=1*
**Antihypertensive drug use**				
Yes	5	1498 / 18637	2359 / 30253	1.00 (0.94, 1.07)
No	5	212 / 3531	298 / 4926	1.01 (0.84, 1.20)
			Heterogeneity:	*unadjusted P=0.786, adjusted P=1*

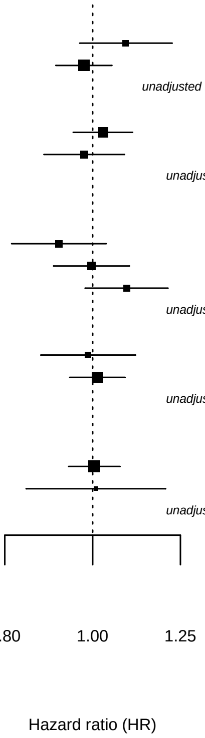

ACEI=angiotensin-converting enzyme inhibitors. ARB=angiotensin-II receptor blockers. BB=ß-blockers. CCB=calcium channel blockers. CI=confidence interval. HR=hazard ratio.

Supplementary Figure 4. Two-stage individual participant-level data meta-analysis for any cancer.

Drug class comparison	Number of events / participants: Treatment	Control	HR (95% CI)
ACEI vs other			
AASK	9 / 436	15 / 658	0.90 (0.39, 2.05)
ABCD	20 / 235	18 / 235	1.22 (0.64, 2.31)
ALLHAT	863 / 9054	2831 / 33364	1.06 (0.99, 1.15)
ANBP2	198 / 3044	192 / 3039	1.02 (0.83, 1.24)
BENEDICT	4 / 301	20 / 903	0.60 (0.20, 1.76)
CAMELOT	34 / 673	78 / 1318	0.84 (0.56, 1.26)
DIABHYCAR	95 / 2443	97 / 2469	1.00 (0.74, 1.35)
EUROPA	223 / 6110	228 / 6108	0.97 (0.81, 1.17)
HOMED−BP	5 / 1172	20 / 2346	0.52 (0.19, 1.38)
HOPE	109 / 4645	106 / 4652	1.01 (0.77, 1.32)
JMIC−B	2 / 822	2 / 828	1.09 (0.15, 7.76)
ONTARGET	759 / 8576	1633 / 17044	0.92 (0.84, 1.00)
PART−2	14 / 308	13 / 309	1.06 (0.50, 2.26)
PREVEND IT	19 / 431	11 / 433	1.77 (0.84, 3.73)
STOP−2	96 / 2205	210 / 4409	0.92 (0.72, 1.17)
All ACE comparison trials	2450 / 40455	5475 / 78115	0.99 (0.95, 1.04)
		Heterogeneity:	I^2= 0%; P=0.554
ARB vs other			
ACTIVE I	446 / 4518	474 / 4498	0.94 (0.83, 1.07)
CASE−J	49 / 2354	46 / 2349	1.11 (0.74, 1.66)
COPE	41 / 1110	89 / 2183	0.90 (0.62, 1.30)
HIJ−CREATE	54 / 1024	62 / 1025	0.89 (0.62, 1.28)
HOMED−BP	9 / 1175	16 / 2343	1.12 (0.49, 2.53)
LIFE	477 / 4605	449 / 4588	1.07 (0.94, 1.22)
MOSES	20 / 681	23 / 671	0.85 (0.47, 1.55)
ONTARGET	787 / 8542	1605 / 17078	0.98 (0.90, 1.06)
PROFESS	340 / 9873	351 / 9925	1.02 (0.87, 1.19)
TRANSCEND	236 / 2954	204 / 2972	1.18 (0.98, 1.43)
VALUE	695 / 7649	816 / 7596	0.85 (0.77, 0.94)
All ARB comparison trials	3154 / 44485	4135 / 55228	0.97 (0.92, 1.01)
		Heterogeneity:	I^2= 33%; P=0.138
BB vs other			
AASK	9 / 441	15 / 653	0.90 (0.39, 2.05)
ASCOT−BPLA	317 / 9618	312 / 9639	1.03 (0.88, 1.21)
COPE	44 / 1089	86 / 2204	1.10 (0.76, 1.58)
ELSA	5 / 1157	7 / 1177	0.75 (0.24, 2.36)
LIFE	447 / 4586	475 / 4603	0.94 (0.82, 1.07)
All BB comparison trials	822 / 16891	895 / 18276	0.98 (0.89, 1.08)
		Heterogeneity:	I^2= 0%; P=0.817
CCB vs other			
AASK	6 / 217	18 / 877	1.34 (0.53, 3.39)
ABCD	18 / 235	20 / 235	0.82 (0.43, 1.56)
ALLHAT	850 / 9048	2844 / 33370	1.04 (0.97, 1.13)
ASCOT−BPLA	312 / 9639	317 / 9618	0.97 (0.83, 1.13)
BENEDICT	6 / 303	18 / 901	1.01 (0.40, 2.54)
CAMELOT	42 / 663	70 / 1328	1.21 (0.83, 1.77)
CASE−J	46 / 2349	49 / 2354	0.90 (0.60, 1.35)
COLM	63 / 2568	80 / 2573	0.78 (0.56, 1.08)
CONVINCE	310 / 8179	299 / 8297	1.06 (0.90, 1.24)
ELSA	7 / 1177	5 / 1157	1.33 (0.42, 4.21)
HOMED−BP	11 / 1171	14 / 2347	1.53 (0.70, 3.38)
INVEST	183 / 10648	168 / 10672	1.10 (0.89, 1.35)
JMIC−B	2 / 828	2 / 822	0.92 (0.13, 6.54)
MOSES	23 / 671	20 / 681	1.18 (0.65, 2.14)
NICS−EH	6 / 204	4 / 210	1.40 (0.39, 4.98)
PREVENT	15 / 417	8 / 408	2.16 (0.88, 5.30)
STOP−2	108 / 2196	198 / 4418	1.09 (0.86, 1.38)
Syst−Eur	183 / 2398	191 / 2297	0.91 (0.74, 1.11)
VALUE	816 / 7596	695 / 7649	1.18 (1.06, 1.30)
All CCB comparison trials	3008 / 60507	5020 / 90214	1.06 (1.02, 1.12)
		Heterogeneity:	I^2= 0%; P=0.492
Thiazide vs other			
ALLHAT	1389 / 15255	2305 / 27163	1.00 (0.94, 1.07)
ANBP2	192 / 3039	198 / 3044	0.98 (0.81, 1.20)
COLM	80 / 2573	63 / 2568	1.29 (0.93, 1.79)
COPE	43 / 1094	84 / 2199	1.02 (0.70, 1.47)
EWPHE	17 / 416	13 / 424	1.29 (0.63, 2.67)
NICS−EH	4 / 210	6 / 204	0.72 (0.20, 2.55)
All thiazide comparison trials	1725 / 22587	2669 / 35602	1.01 (0.95, 1.07)
		Heterogeneity:	I^2= 0%; P=0.702

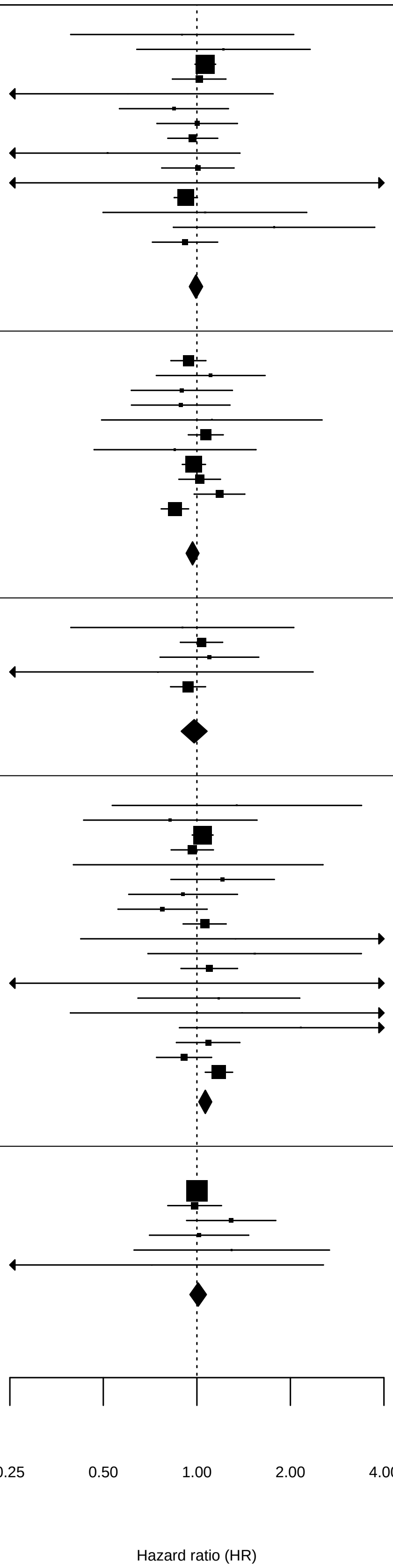

ACEI=angiotensin-converting enzyme inhibitors. ARB=angiotensin-II receptor blockers. BB=ß-blockers. CCB=calcium channel blockers. CI=confidence interval. HR=hazard ratio. Trial name acronyms are described in full in the footnote of **Supplementary Table 3.**

Supplementary tables

Supplementary Table 1. MEDLINE search strategy for BPLTTC.

Search (((((("Hypertension/drug effects"[Mesh] OR "Hypertension/drug therapy"[Mesh]))) AND (("Blood Pressure/drug effects"[Mesh] OR "Blood Pressure/therapy"[Mesh]))) AND ((Clinical Trial[ptyp] OR Controlled Clinical Trial[ptyp] OR Meta-Analysis[ptyp] OR Randomized Controlled Trial[ptyp] OR Clinical Trial, Phase III[ptyp]) AND Humans[Mesh] AND adult[MeSH]))) AND (((((((((("Antihypertensive Agents" [Pharmacological Action]) OR "Antihypertensive Agents/therapeutic use"[Mesh])) OR ((("Vasodilator Agents" [Pharmacological Action])) OR ("Vasodilator Agents/therapeutic use"[Mesh] OR "Vasodilator Agents/therapy"[Mesh]))) OR (("Adrenergic alpha-Antagonists/therapeutic use"[Mesh]) OR "Adrenergic alpha-Antagonists" [Pharmacological Action])) OR (("Adrenergic beta-Antagonists" [Pharmacological Action]) OR "Adrenergic beta-Antagonists/therapeutic use"[Mesh])) OR (("Sodium Chloride Symporter Inhibitors" [Pharmacological Action]) OR "Sodium Chloride Symporter Inhibitors/therapeutic use"[Mesh])) OR "Angiotensin-Converting Enzyme Inhibitors/therapeutic use"[Mesh]) OR (("Angiotensin II Type 1 Receptor Blockers" [Pharmacological Action]) OR "Angiotensin II Type 1 Receptor Blockers/therapeutic use"[Mesh])) OR (("Calcium Channel Blockers" [Pharmacological Action]) OR "Calcium Channel Blockers/therapeutic use"[Mesh])) AND ((Clinical Trial[ptyp] OR Controlled Clinical Trial[ptyp] OR Meta-Analysis[ptyp] OR Randomized Controlled Trial[ptyp] OR Clinical Trial, Phase III[ptyp]) AND Humans[Mesh] AND adult[MeSH])) Filters: Clinical Trial; Controlled Clinical Trial; Randomized Controlled Trial; Clinical Trial, Phase III; Meta-Analysis; Systematic Reviews; Humans; Adult: 19+ years

BPLTTC=Blood Pressure Lowering Treatment Trialists' Collaboration.

Supplementary Table 2. Full list of variables requested from participating trials.

Type of data	Variables
**Study-level**	Region
	Treatment and comparator groups
	Study period/duration of follow-up
	Randomization method
	Outcome ascertainment
	Early stopping and reasons
	Funding source
**Participant-level**	
Baseline information	Patient Identifier
	Sex
	Date of birth *or* age at randomization
	Ethnicity
	History of diabetes mellitus
	Currently treated hypertension
	History of chronic kidney disease
	History of cardiovascular disease (coronary heart and cerebrovascular disease)
	History of peripheral vascular disease
	History of atrial fibrillation
	Height
	Weight
	Systolic blood pressure
	Diastolic blood pressure
	Smoking status
	Estimated alcohol intake
	**Baseline drug therapy:**
	Lipid lowering therapy at baseline
	Antiplatelet or anticoagulant therapy
	Antihypertensives (ACEIs, ARBs, BBs, CCBs, diuretics, α-blockers)
	**Randomization blood and urine measurements:**
	Haemoglobin concentration
	Fasting blood glucose
	C-reactive protein
	Serum albumin
	Baseline total cholesterol
	Baseline HDL cholesterol
	Serum/plasma creatinine
	Urinary albumin/protein excretion or concentration
	Albuminuria/proteinuria (if quantitative measure for excretion/concentration not available)
Randomization data and scheduled follow-up	Date of randomization
	Randomized treatment allocation code
	Scheduled end-date of trial treatment
	Date of last follow-up
Outcomes (diagnosed after randomization)	Stroke (date, outcome (i.e. fatal/non-fatal), confirmation)
	Myocardial infarction (date, outcome)
	Coronary revascularization (date)
	Heart failure leading to hospitalization or death (date, outcome)
	Need for renal replacement therapy (dialysis or transplant) (date)
	Primary site of first cancer diagnosed after randomization (date, outcome)
	First fracture after randomization (date)
	Study treatment stopped early (date, reason)
	Death (date, cause)
	Diabetes diagnosed after randomization (date)
	Retinopathy diagnosed after randomization (date)
	Dementia diagnosed after randomization (date)
	Peripheral vascular disease diagnosed after randomization (date)

Follow-up measurements (repeated for each follow-up visit)	Visit number (n)
	Date of visit
	Weight
	Systolic blood pressure
	Diastolic blood pressure
	Serum/plasma creatinine
	Urinary albumin/protein excretion or concentration
	Visit number (n)
Safety and less common efficacy outcomes after randomization	Name of serious adverse event(s) recorded
	Date of diagnosis of serious adverse event
	Discontinuation of medication
	Date of discontinuation
	Discontinuation due to adverse event (y/n)
	Date of discontinuation
	Acute kidney injury/renal failure recorded
	Date of acute kidney injury/renal failure
	Atrial fibrillation event recorded
	Date of atrial fibrillation event
	Albuminuria/microalbuminuria (new or worsening) event recorded
	Date of albuminuria/microalbuminuria event
	Name of any other adverse event/outcome reported
	Date of adverse event/outcome

ACEI=angiotensin-converting enzyme inhibitors. ARB=angiotensin-II receptor blockers. BB=ß-blockers. CCB=calcium channel blockers. HDL=high-density lipoprotein.

Supplementary Table 3. Characteristics of individual trials included in study.

Trial	Country	Recruitment period	Randomisation groups	Number of participants (% women)	Additional (open-label) treatment	Follow-up duration (year)	Explicit exclusion of cancer patients at baseline	Source of cancer outcomes	Adjudication	Level of detail of cancer outcomes provided
AASK [3,4]	USA	Feb 1995 to Sept 1998	All	1094 (39)	Furosemide, doxazosin, clonidine, hydralazine and minoxidil (sequentially)	4.8	No	Routine adverse event	No	Site of cancer diagnosis (lung, colon, breast, prostate, skin, other types)
			Drug class comparison							
			ACEI (Ramipril)	436 (39)						
			CCB (Amlodipine)	217 (39)						
			β-blocker (Metoprolol)	441 (39)						
			BP-lowering intensity comparison							
			More intense BP-lowering	540 (38)						
			Less intense BP-lowering	554 (40)						
ABCD [5–7]	USA	Mar 1991 to May 1993	All	950 (33)	β-blocker (Metoprolol), diuretic (HCTZ), or others but not CCB or ACEI	4.7	Yes (patients with active cancer)	Routine adverse event	No	Cancer diagnosis yes/no (no information on site of cancer)
			Drug class comparison							
			CCB (Nisoldipine)	235 (32)						
			ACEI (Enalapril)	235 (33)						
			BP-lowering intensity comparison							
			More intense BP-lowering	474 (40)						
			Less intense BP-lowering	476 (38)						
ACTIVE I [8]	Multi-country	Jun 2003 to May 2006	All	9016 (39)	None	4.1	No	Routine adverse event	No	Site of cancer diagnosis (lung, colon, breast, prostate, skin, other types)
			ARB (Irbesartan)	4518 (39)						
			Placebo	4498 (39)						
ALLHAT [9,10]	Multi-country	Feb 1994 to Jan 1998	All	42,418 (47)	Atenolol, clonidine or reserpine	4.8	No	Pre-specified safety outcome	Yes	Site of cancer diagnosis (lung, colon, breast, prostate, bladder, other)
			Diuretic (Chlorthalidone)	15,255 (47)						
			CCB (Amlodipine)	9048 (47)						
			ACEI (Lisinopril)	9054 (46)						
			α-blocker (Doxazosin)	9061 (46)						
ANBP2 [11]	Australia	April 1995 to Jun 1998	All	6083 (51)	β-blocker, CCB and α-blocker	4.1	No	Pre-specified safety outcome	Yes	ICD-9 codes for site of cancer
			ACEI (Enalapril)	3044 (50)						
			Diuretic (HCTZ)	3039 (52)						
ASCOT-BPLA [12,13]	Multi-country	Feb 1998 to May 2000	All	19,257 (23)	For CCB arm: plus ACEI (Perindopril); For β-blocker arm: plus diuretic (Bendroflumethiazide) and potassium	5.3	No	Routine adverse event	No	Fatal cancer yes/ no (no information on site of cancer)
			CCB (Amlodipine-based)	9639 (23)						
			β-blocker (Atenolol-based)	9618 (23)						

BENEDICT [14,15]	Italy	Around 2000 to 2003	All	1204 (47)	Diuretic (HCTZ or furosemide), then doxazosin, prazosin, clonidine, methyldopa or β-blocker, then minoxidil, or CCB	3.1	Yes	Routine adverse event	No	Site of cancer diagnosis (lung, colon, breast, prostate, other)
			ACEI (Trandolapril)	301 (48)						
			CCB (Verapamil)	303 (46)						
			ACEI (Trandolapril) and CCB (Verapamil)	300 (45)						
			Placebo	300 (50)						
CAMELOT [16]	Multi-country	Apr 1999 to Apr 2002	All	1991 (26)	Allowed to continue β-blocker, α-blocker, diuretic	1.6	No	Routine adverse event	No	Site of cancer diagnosis (text description)
			CCB (Amlodipine)	663 (24)						
			ACEI (Enalapril)	673 (28)						
			Placebo	655 (27)						
CASE-J [17,18]	Japan	Sep 2001 to Jan 2003	All	4703 (45)	Allowed to continue background treatment (diuretic, α-blocker, β-blocker); Can add other except ARB, CCB, ACEI	3.1	Yes (patients with history of malignant tumour (confirmed or suspected) within 5 years of enrolment)	Routine adverse event	No	Site of cancer diagnosis (text description)
			ARB (Candesartan)	2354 (46)						
			CCB (Amlodipine)	2349 (43)						
COLM [19,20]	Japan	Apr 2007 to Sep 2008	All	5141 (48)	β-blocker, α-blocker, ACEI	3.0	Yes (patients with malignant tumours)	Routine adverse event	Yes	Cancer diagnosis yes/no (no information on site of cancer)
			CCB (Amlodipine or azelnidipine) and ARB (Olmesartan)	2568 (48)						
			Diuretic (HCTZ, Trichlormethiazide, or indapamide) and ARB (Olmesartan)	2573 (48)						
CONVINCE [21,22]	Multi-country	Sep 1996 to Dec 1998	All	16476 (55)	Additional treatment if necessary	2.8	Yes (disease likely to cause death within 5 years of enrolment, e.g. untreated malignancy)	Pre-specified safety outcome	Yes	Cancer diagnosis yes/no (no information on site of cancer)
			CCB (Verapamil)	8179 (56)						
			β-blocker (Atenolol) or diuretic (HCTZ)	8297 (56)						
COPE [23]	Japan	Jun 2003 to Nov 2006	All	3293 (49)	Additional treatment if necessary	3.6	Yes (history of malignancy 5 years prior to study entry)	Routine adverse event	No	MedDRA codes for site of cancer
			ARB/CCB (ARB/Benidipine)	1110 (49)						
			β-blocker/CCB (β-blocker/Benidipine)	1089 (49)						
			Diuretic/CCB (Thiazide/Benidipine)	1094 (49)						
DIABHYCAR [24,25]	The Nether-lands	Oct 1997 to Jun 2000	All	4912 (30)	Usual treatment	3.9	Yes (patients with poor life expectancy, e.g. due to cancer)	Routine adverse event	No	Site of cancer diagnosis (text description)
			ACEI (Ramipril)	2443 (30)						
			Placebo	2469 (30)						

ELSA [26,27]	Multi-country	Possibly between 1994 to 1998	All<br>CCB (Lacidipine)<br>β-blocker (Atenolol)	2334 (45)<br>1177 (46)<br>1157 (45)	Diuretic (HCTZ)	3.4	No	Routine adverse event	No	ICD-9 codes for site of fatal cancer
EUROPA [28,29]	Multi-country (Europe)	Oct 1997 to Jun 2000	All<br>ACEI (Perindopril)<br>Placebo	12,218 (15)<br>6110 (14)<br>6108 (15)	None specified	4.2	No	Routine adverse event	No	Site of cancer diagnosis (text description)
EWPHE [30,31]	Multi-country	From 1972	All<br>Diuretic (HCTZ or triamterene)<br>Placebo	840 (70)<br>416 (69)<br>424 (71)	Methyldopa	4.6	Yes (malignancy)	Routine adverse event	No	ICD-8 codes for site of cancer
HIJ-CREATE [32]	Japan	Jun 2001 to Apr 2004	All<br>ARB (Candesartan)<br>Non-ARB (including ACEI)	2049 (20)<br>1024 (18)<br>1025 (21)	None	4.0	Yes (known malignant neoplasm)	Routine adverse event	Yes	Site of cancer diagnosis (text description)
HOMED-BP [33]	Japan	May 2001 to Oct 2009	All<br>*Drug class comparison*<br>ACEI<br>ARB<br>CCB<br>*BP-lowering intensity comparison*<br>More intense BP-lowering<br>Less intense BP-lowering	3518 (50)<br><br>1172 (50)<br>1175 (50)<br>1171 (50)<br><br>1759 (50)<br>1759 (50)	Diuretic; β-blocker; then other drugs (avoid reaching BP <110/65 mmHg)	4.9	No	Routine adverse event	No	ICD-10 codes for site of fatal cancer
HOPE [34]	Multi-country	Dec 1993 to Jun 1995	All<br>ACEI (Ramipril)<br>Placebo	9297 (27)<br>4656 (28)<br>4652 (26)	None specified	4.5	No	Pre-specified safety outcome	Yes	Cancer diagnosis yes/no (no information on site of cancer)
INVEST [35]	Multi-country	From Jan 1998	All<br>CCB (Verapamil)<br>Non-CCB (Atenolol)	21,230 (52)<br>10,648 (52)<br>10,672 (52)	ACEI (Trandolapril) and/or diuretic (HCTZ)	2.8	Yes (but patients with history of skin, prostate and other cancer with long survival expectancy were not necessarily excluded)	Pre-specified safety outcome	Yes	Site of cancer diagnosis (lung, colon, breast, prostate, bladder, other)
JMIC-B [36]	Japan	Jan 1994 to Jul 1997	All<br>CCB (Nifedipine)<br>ACEI (Enalapril, imidapril or lisinopril)	1650 (31)<br>828 (32)<br>822 (30)	α-blocker (doxazosin, bunazosin or prazosin); nitrates or β-blocker for angina if needed	2.3	No	Pre-specified safety outcome	Yes	ICD-9 codes for site of fatal cancer
LIFE [37,38]	Multi-country	June 1995 to May 1997	All<br>ARB (Losartan)<br>β-blocker (Atenolol)	9193 (54)<br>4605 (54)<br>4588 (54)	Diuretic (HCTZ) and other except ACEI, ARB and β-blocker	4.9	No	Pre-specified safety outcome	No	Site of cancer diagnosis (text description)

MOSES [39]	Germany and Austria	Oct 1998 to Feb 2002	All	1352 (46)	Diuretic, β-blocker, α-blocker or centrally-acting drugs; ACEI, ARB or CCB only if clinically necessary	3.3	No	Routine adverse event	Yes	ICD-10 codes for site of cancer
			ARB (Eprosartan)	681 (46)						
			CCB (Nitrendipine)	671 (45)						
NICS-EH [40]	Japan	Oct 1989 to Apr 1992	All	414 (67)	Titration but no additional treatment	3.2	No	Pre-specified safety outcome	Yes	Site of cancer diagnosis (lung, bowel, breast, other)
			CCB (Nicardipine)	204 (60)						
			Diuretic (Trichlormethiazide)	210 (74)						
ONTARGET [41,42]	Multi-country	Jan 2002 to Aug 2003	All	25,620 (27)	None	4.8	No	Pre-specified safety outcome	No	Site of cancer diagnosis (lung, colon, breast, prostate, other)
			ACEI (Ramipril)	8576 (27)						
			ARB (Telmisartan)	8542 (26)						
			ACEI (Ramipril) and ARB (Telmisartan)	8502 (26)						
PART-2 [43]	New Zealand	Not specified; Publicat-ion in 2000	All	617 (18)	None	4.6	No	Routine adverse event	No	Site of cancer diagnosis (lung, colon, breast, other)
			ACEI (Ramipril)	308 (18)						
			Placebo	309 (18)						
PREVEND IT [44]	The Nether-lands	Apr 1998 to Jun 1999	All	864 (35)	None	3.8	No	Routine adverse event	No	ICD-10 codes for site of fatal cancer
			ACEI (Fosinopril)	433 (36)						
			Placebo	431 (34)						
PREVENT [45,46]	USA and Canada	Nov 1992 to Sep 1994	All	825 (20)	None	3.0	No	Pre-specified safety outcome	Yes	Site of cancer diagnosis (text description)
			CCB (Amlodipine)	417 (20)						
			Placebo	408 (20)						
PROFESS [47,48]	Multi-country	Sep 2003 to Jul 2006	All	19,798 (36)	At physician's discretion to control blood pressure: diuretic, then β-blocker or CCB, then ACEI but not ARB	2.5	Yes	Pre-specified safety outcome	Yes	Site of cancer diagnosis (text description)
			ARB (Telmisartan)	9873 (35)						
			Placebo	9925(36)						
STOP Hyper-tension-2 [49]	Sweden	Sep 1992 to Dec 1994 1987 to 1991	All	6614 (67)		4.5	No	Routine adverse event	No	Site of cancer diagnosis (text description)
			Conventional: β-blocker (Atenolol or metoprolol), diuretic (HCTZ) or both	2213 (68)						
			ACEI (Enalapril or lisinopril	2205 (66)						
			CCB (Felodipine or isradipine)	2196 (66)						
Syst-Eur [50]	Multi-country	Dec 1988 to Jan 1997	All	4695 (67)	ACEI (Enalapril) and/or diuretic (HCTZ)	2.6	No	Pre-specified safety outcome	Yes	Site of cancer diagnosis (lung, colon, breast, prostate, other)
			CCB (Nitrendipine)	2398 (67)						
			Placebo	2297 (66)						

TRANSCEND [41,51]	Multi-country	Nov 2001 to May 2004	All<br>ARB (Telmisartan)<br>Placebo	5926 (43)<br>2954 (43)<br>2972 (43)	None	4.9	No	Pre-specified safety outcome	No	Site of cancer diagnosis (lung, colon, breast, prostate, other)
VALUE [52,53]	Multi-country	Sept 1997 to Dec 1999	All<br>ARB (Valsartan-based)<br>CCB (Amlodipine-based)	15,245 (42)<br>7649 (42)<br>7596 (42)	Diuretic (HCTZ, then other antihypertensive drugs except ARB (ACEI or CCB if clinically indicated other than for hypertension)	4.2	No	Routine adverse event	No	MedDRA codes for site of cancer

AASK=African American Study of Kidney Disease and Hypertension. ABCD=Appropriate Blood Pressure Control in Diabetes. ACEI=angiotensin-converting enzyme inhibitors. ACTIVE I=Atrial Fibrillation Clopidogrel Trial with Irbesartan for Prevention of Vascular Events. ALLHAT=Antihypertensive and Lipid Lowering Treatment to Prevent Heart Attack Trial. ANBP2=Second Australian National Blood Pressure Study. ARB=angiotensin-II receptor blockers. ASCOT-BPLA=Anglo-Scandinavian Cardiac Outcomes Trial-Blood Pressure Lowering Arm. BB=ß-blockers. BENEDICT=BErgamo NEphrologic DIabetes Complications Trial. CAMELOT=Comparison of Amlodipine vs Enalapril to Limit Occurrences of Thrombosis. CASE-J=Candesartan Antihypertensive Survival Evaluation in Japan Trial. CCB=calcium channel blockers. COLM=Combination of OLMesartan study. CONVINCE=Controlled ONset Verapamil INvestigation of Cardiovascular Endpoints trial. COPE=Combination Therapy of Hypertension to Prevent Cardiovascular Events. DIABHYCAR=Noninsulin-dependent diabetes, hypertension, microalbuminuria or proteinuria, cardiovascular events, and ramipril. ELSA=European Lacidipine Study on Atherosclerosis. EUROPA=EUropean trial on Reduction Of cardiac events with Perindopril in patients with stable coronary Artery disease. EWPHE=European Working Party on High Blood Pressure in the Elderly. HCTZ=hydrochlorothiazide. HIJ-CREATE=Heart Institute of Japan Candesartan Randomized Trial for Evaluation in Coronary Artery Disease. HOMED-BP=Hypertension Objective Treatment Based on Measurement by Electrical Devices of Blood Pressure. HOPE=Heart Outcomes Prevention Evaluation. ICD=International Classification of Diseases. INVEST=International Verapamil-Trandolapril Study. JMIC-B=Japan Multicenter Investigation for Cardiovascular Diseases-B. LIFE=Losartan Intervention For Endpoint reduction. MedDRA=Medical Dictionary for Regulatory Activities. MOSES=Morbidity and Mortality After Stroke, Eprosartan Compared With Nitrendipine for Secondary Prevention. NICS-EH=National Intervention Cooperative Study in Elderly Hypertensives. ONTARGET=Ongoing Telmisartan Alone and in Combination with Ramipril Global Endpoint Trial. PART-2=Prevention of Atherosclerosis with Ramipril Trial. PREVEND IT=Prevention of Renal and Vascular Endstage Disease Intervention Trial. PREVENT=Prospective Randomized Evaluation of the Vascular Effects of Norvasc Trial. PROFESS=Prevention Regimen for Effectively Avoiding Second Strokes. STOP Hypertension 2=Swedish Trial in Old Patients with Hypertension-2. Syst-Eur=Systolic Hypertension in Europe. TRANSCEND=Telmisartan Randomized Assessment Study in ACE Intolerant Subjects with Cardiovascular Disease. VALUE=Valsartan Antihypertensive Long-term Use Evaluation.

Supplementary Table 4. List of trials and interventions assigned to the trial arms.

Trial	Active group	Control group(s)
**ACEI vs other comparisons (15 trials)**		
AASK [3,4]	ACEI	ß-blocker, CCB
ABCD [5–7]	ACEI	CCB
ALLHAT [9,10]	ACEI	CCB, thiazide, α-blocker
ANBP2 [11]	ACEI	Thiazide
BENEDICT [14,15]	ACEI	ACEI/CCB, CCB, placebo
CAMELOT [16]	ACEI	CCB, placebo
DIABHYCAR [24,25]	ACEI	Placebo
EUROPA [28,29]	ACEI	Placebo
HOMED-BP [33]	ACEI	ARB, CCB
HOPE [34]	ACEI	Placebo
JMIC-B [36]	ACEI	CCB
ONTARGET [41,42]	ACEI	ACEI/ARB, ARB
PART-2 [43]	ACEI	Placebo
PREVEND IT [44]	ACEI	Placebo
STOP Hypertension-2 [49]	ACEI	ß-blocker and/or thiazide, CCB
**ARB vs other comparisons (11 trials)**		
ACTIVE I [8]	ARB	Placebo
CASE-J [17,18]	ARB	CCB
COPE [23]	ARB (/CCB)	CCB/ß-blocker, CCB/thiazide
HIJ-CREATE [32]	ARB	Non-ARB
HOMED-BP [33]	ARB	ACEI, CCB
LIFE [37,38]	ARB	ß-blocker
MOSES [39]	ARB	CCB
ONTARGET [41,42]	ARB	ACEI, ACEI/ARB
PROFESS [47,48]	ARB	Placebo
TRANSCEND [41,51]	ARB	Placebo
VALUE [52,53]	ARB-based	CCB-based
**BB vs other comparisons (5 trials)**		
AASK [3,4]	ß-blocker	ACEI, CCB
ASCOT-BPLA [12,13]	ß-blocker-based	CCB-based
COPE [23]	ß-blocker (/CCB)	CCB/ARB, CCB/thiazide
ELSA [26,27]	ß-blocker	CCB
LIFE [37,38]	ß-blocker	ARB
**CCB vs other comparisons (19 trials)**		
AASK [3,4]	CCB	ACEI, ß-blocker
ABCD [5–7]	CCB	ACEI
ALLHAT [9,10]	CCB	ACEI, thiazide, α-blocker
ASCOT-BPLA [12,13]	CCB-based	ß-blocker-based
BENEDICT [14,15]	CCB	ACEI, ACEI/CCB, placebo
CAMELOT [16]	CCB	ACEI, placebo
CASE-J [17,18]	CCB	ARB
COLM [19,20]	CCB (/ARB)	ARB/thiazide
CONVINCE [21,22]	CCB	ß-blocker/thiazide
ELSA [26,27]	CCB	ß-blocker
HOMED-BP [33]	CCB	ACEI, ARB
INVEST [35]	CCB	Non-CCB
JMIC-B [36]	CCB	ACEI
MOSES [39]	CCB	ARB
NICS-EH [40]	CCB	Thiazide
PREVENT [45,46]	CCB	Placebo
STOP Hypertension-2 [49]	CCB	ACEI, ß-blocker and/or thiazide
Syst-Eur [50]	CCB	Placebo
VALUE [52,53]	CCB-based	ARB-based

**Thiazide diuretic vs other comparisons (6 trials)**		
ALLHAT [9,10]	Thiazide	ACEI, CCB, α-blocker
ANBP2 [11]	Thiazide	ACEI
COLM [19,20]	Thiazide (/ARB)	ARB/CCB
COPE [23]	Thiazide (/CCB)	CCB/ARB and CCB/ß-blocker
EWPHE [30,31]	Thiazide	Placebo
NICS-EH [40]	Thiazide	CCB

ACEI=angiotensin-converting enzyme inhibitors. ARB=angiotensin-II receptor blockers. BB=ß-blockers. CCB=calcium channel blockers. Trial name acronyms are described in full in the footnote of **Supplementary Table 3.**

Supplementary Table 5. Risk of bias assessment for individual trials.

Trial	Risk of bias arising from randomization	Risk of bias due to effect of assignment to intervention	Risk of bias due to missing outcome data	Risk of bias due to measurement of outcome	Risk of bias due to reporting of result	Overall risk of bias
AASK [3,4]	Low	Low	Low	Low	Low	Low
ABCD [5–7]	Low	Low	Some	Low	Low	Low
ACTIVE I [8]	Low	Low	Some	Low	Low	Low
ALLHAT [9,10]	Low	Low	Low	Low	Low	Low
ANBP2 [11]	Low	Some	Some	Low	Low	Some
ASCOT-BPLA [12,13]	Low	Some	Low	Low	Low	Low
BENEDICT [14,15]	Low	Low	Low	Low	Low	Low
CAMELOT [16]	Low	Low	Low	Low	Low	Low
CASE-J [17,18]	Low	Some	Low	Low	Low	Low
COLM [19,20]	Low	Some	Low	Low	Low	Low
CONVINCE [21,22]	Low	Low	Low	Low	Low	Low
COPE [23]	Low	Some	Some	Low	Low	Some
DIABHYCAR [24,25]	Low	Low	Low	Low	Low	Low
ELSA [26,27]	Low	Low	Low	Low	Low	Low
EUROPA [28,29]	Low	Low	Low	Low	Low	Low
EWPHE [30,31]	Low	Low	Low	Low	Low	Low
HIJ-CREATE [32]	Low	Some	Low	Low	Low	Low
HOMED-BP [33]	Low	Some	Some	Low	Low	Some
HOPE [34]	Low	Some	Low	Low	Low	Low
INVEST [35]	Low	Some	Some	Low	Low	Some
JMIC-B [36]	Low	Some	Low	Low	Low	Low
LIFE [37,38]	Low	Low	Low	Low	Low	Low
MOSES [39]	Low	Some	Low	Low	Low	Low
NICS-EH [40]	Low	Some	Low	Low	Low	Low
ONTARGET [41,42]	Low	Some	Low	Low	Low	Low
PART-2 [43]	Low	Low	Low	Low	Low	Low
PREVEND IT [44]	Low	Low	Some	Low	Low	Low
PREVENT [45,46]	Low	Low	Low	Low	Low	Low
PROFESS [47,48]	Low	Low	Low	Low	Low	Low
STOP Hypertension-2 [49]	Low	Some	Low	Low	Low	Low
Syst-Eur [50]	Low	Some	Low	Low	Low	Low
TRANSCEND [41,51]	Low	Low	Low	Low	Low	Low
VALUE [52,53]	Low	Low	Low	Low	Low	Low

Trial name acronyms are described in full in the footnote of **Supplementary Table 3.**

Supplementary Table 6. Characteristics of participants at baseline for each trial.

Trial	Participants, N (% women)	Age (years), mean (SD)	Body mass index (kg/m^2), mean (SD)	% Current smokers (N)
AASK [3,4]	1094 (39)	54 (11)	30.6 (6.6)	29 (321)
ABCD [5–7]	470 (39)	58 (8)	31.7 (5.7)	14 (128)
ACTIVE I [8]	9016 (39)	70 (10)	29.1 (5.8)	8 (698)
ALLHAT [9,10]	42,418 (47)	67 (8)	29.6 (5.9)	22 (9269)
ANBP2 [11]	6083 (51)	73 (5)	27.1 (4.2)	7 (431)
ASCOT-BPLA [12,13]	19,257 (23)	63 (9)	28.7 (4.6)	33 (6277)
BENEDICT [14,15]	1204 (47)	62 (8)	29.1 (4.7)	12 (146)
CAMELOT [16]	1991 (26)	58 (10)	29.8 (5.3)	26 (528)
CASE-J [17,18]	4703 (45)	64 (11)	24.5 (3.7)	22 (1025)
COLM [19,20]	5141 (48)	74 (5)	24.3 (3.4)	11 (551)
CONVINCE [21,22]	16,476 (56)	66 (7)	-	23 (3795)
COPE [23]	3293 (49)	64 (11)	24.5 (3.4)	21 (700)
DIABHYCAR [24,25]	4912 (30)	65 (8)	29.2 (4.6)	15 (756)
ELSA [26,27]	2334 (46)	57 (7)	27.2 (3.8)	20 (478)
EUROPA [28,29]	12,218 (15)	61 (9)	27.4 (3.5)	15 (1862)
EWPHE [30,31]	840 (70)	71 (8)	26.4 (4.5)	17 (143)
HIJ-CREATE [32]	2049 (20)	65 (9)	24.6 (3)	25 (509)
HOMED-BP [33]	3518 (50)	60 (10)	24.4 (3.5)	21 (743)
HOPE [34]	9297 (27)	66 (7)	27.7 (4.4)	14 (1319)
INVEST [35]	21,320 (51)	66 (10)	29.2 (7.1)	12 (2809)
JMIC-B [36]	1650 (31)	65 (85)	24 (2.9)	34 (563)
LIFE [37,38]	9193 (54)	67 (7)	28 (4.8)	16 (1499)
MOSES [39]	1352 (46)	68 (10)	27.5 (4.3)	18 (247)
NICS-EH [40]	414 (67)	70 (7)	23.4 (3.1)	9 (38)
ONTARGET [41,42]	25,620 (27)	67 (7)	28.2 (4.8)	13 (3225)
PART-2 [43]	617 (18)	60 (8)	26.8 (3.6)	16 (100)
PREVEND IT [44]	864 (35)	51 (12)	26.4 (4.4)	40 (345)
PREVENT [45,46]	825 (20)	57 (10)	28 (4.8)	25 (204)
PROFESS [47,48]	19,798 (36)	66 (8)	26.8 (5)	21 (4231)
STOP Hypertension-2 [49]	6614 (67)	76 (4)	26.7 (4)	9 (594)
Syst-Eur [50]	4695 (67)	70 (7)	27 (4.1)	7 (343)
TRANSCEND [41,51]	5926 (43)	68 (7)	28.2 (4.8)	10 (582)
VALUE [52,53]	15,245 (42)	67 (8)	28.6 (5)	24 (3664)

SD=standard deviation. Trial name acronyms are described in full in the footnote of **Supplementary Table 3.**

Supplementary Table 7. Effects of antihypertensive drug classes on the risk of any cancer and cancer death, based on direct comparison and network meta-analysis estimates.

	Any cancer		Cancer death	
	**Direct comparison**	**Network estimate**	**Direct comparison**	**Network estimate**
**Placebo**	1.0 (reference)	1.0 (reference)	1.0 (reference)	1.0 (reference)
ACEI	1.00 (0.88 – 1.13)	1.00 (0.93 – 1.09)	1.09 (0.85 – 1.40)	1.02 (0.86 – 1.22)
ARB	1.00 (0.92 – 1.09)	0.99 (0.92 – 1.06)	0.98 (0.81 – 1.19)	0.98 (0.83 – 1.14)
BB		0.99 (0.89 – 1.11)		1.00 (0.80 – 1.24)
CCB	0.98 (0.82 – 1.17)	1.04 (0.96 – 1.13)	0.69 (0.39 – 1.21)	0.97 (0.80 – 1.18)
Thiazide	1.29 (0.63 – 2.65)	1.00 (0.90 – 1.10)		1.05 (0.86 – 1.29)
**ACEI**	1.0 (reference)	1.0 (reference)	1.0 (reference)	1.0 (reference)
ARB	1.05 (0.95 – 1.16)	0.99 (0.92 – 1.05)	0.99 (0.82 – 1.20)	0.95 (0.82 – 1.10)
BB	1.00 (0.40 – 2.50)	0.99 (0.89 – 1.09)	0.88 (0.32 – 2.42)	0.97 (0.82 – 1.16)
CCB	1.01 (0.93 – 1.10)	1.04 (0.97 – 1.11)	0.96 (0.83 – 1.12)	0.95 (0.83 – 1.09)
Thiazide	0.95 (0.88 – 1.03)	0.99 (0.92 – 1.07)	1.03 (0.90 – 1.16)	1.02 (0.90 – 1.16)
**ARB**	1.0 (reference)	1.0 (reference)	1.0 (reference)	1.0 (reference)
BB	0.95 (0.84 – 1.08)	1.00 (0.91 – 1.10)	1.04 (0.81 – 1.35)	1.02 (0.85 – 1.22)
CCB	1.16 (1.06 – 1.28)	1.05 (0.99 – 1.12)	1.37 (0.67 – 2.78)	1.00 (0.84 – 1.18)
Thiazide	1.10 (0.71 – 1.69)	1.01 (0.92 – 1.10)		1.08 (0.90 – 1.29)
**BB**	1.0 (reference)	1.0 (reference)	1.0 (reference)	1.0 (reference)
CCB	1.01 (0.89 – 1.14)	1.05 (0.96 – 1.15)	0.98 (0.84 – 1.15)	0.98 (0.85 – 1.12)
Thiazide	0.96 (0.63 – 1.47)	1.01 (0.90 – 1.13)		1.05 (0.88 – 1.26)
**CCB**	1.0 (reference)	1.0 (reference)	1.0 (reference)	1.0 (reference)
Thiazide	0.99 (0.90 – 1.08)	0.96 (0.89 – 1.03)	1.07 (0.93 – 1.23)	1.08 (0.94 – 1.23)

ACEI=angiotensin-converting enzyme inhibitors. ARB=angiotensin-II receptor blockers. BB=ß-blockers. CCB=calcium channel blockers.

Supplementary Table 8. Effects of antihypertensive drug classes compared against all other comparators on any cancer stratified by explicit exclusion of cancer patients at baseline.

	**Exclusion of cancer patients at baseline**			**No explicit exclusion of cancer patients at baseline**			
	N trials	N events (treatment/ comparison)	HR (95% CI)	N trials	N events (treatment/ comparison)	HR (95% CI)	Heterogeneity
**ACEI**	3	119 / 135	0.99 (0.76 – 1.28)	12	2331 / 5340	0.99 (0.94 – 1.04)	P = 0.99
**ARB**	4	484 / 548	0.98 (0.86 – 1.11)	7	2670 / 3587	0.96 (0.91 – 1.01)	P = 0.78
**BB**	1	44 / 86	1.09 (0.76 – 1.57)	4	778 / 809	0.97 (0.88 – 1.07)	P = 0.55
**CCB**	6	626 / 634	1.02 (0.91 – 1.14)	13	2382 / 4386	1.07 (1.02 – 1.13)	P = 0.40
**Thiazide**	3	142 / 161	1.18 (0.94 – 1.48)	3	1585 / 2509	0.99 (0.93 – 1.06)	P = 0.17

ACEI=angiotensin-converting enzyme inhibitors. ARB=angiotensin-II receptor blockers. BB=ß-blockers. CCB=calcium channel blockers. CI=confidence interval. HR=hazard ratio.

Collaborating Trialists

A Adler (UKPDS [54] [UK Prospective Diabetes Study]),
L Agodoa (AASK [3,4] [African-American Study of Kidney Disease and Hypertension]),
A Algra (Dutch TIA Trial [55] [Dutch Transient Ischemic Attack Trial]),
F W Asselbergs (PREVEND IT [44] [Prevention of Renal and Vascular End- stage Disease Intervention Trial]),
N Beckett (HYVET [56] [Hypertension in the Very Elderly Trial]),
E Berge (deceased) (VALUE [52,53] trial [Valsartan Antihypertensive Long-term Use Evaluation trial]),
H Black (CONVINCE [21,22] [Controlled Onset Verapamil Investigation of Cardiovascular End Points]),
F P J Brouwers (PREVEND IT [44]),
M Brown (INSIGHT [57] [International Nifedipine GITS Study: Intervention as a Goal in Hypertension]),
C J Bulpitt (EWPHE [30,31] [European Working Party on High Blood Pressure in the Elderly], HYVET [56]),
B Byington (PREVENT [45,46] [Prospective Randomized Evaluation of the Vascular Effects of Norvasc Trial]),
J Chalmers (ADVANCE [58] [Action in Diabetes and Vascular Disease: Preterax and Diamicron MR Controlled Evaluation], PROGRESS [59] [Perindopril protection against recurrent stroke]),
J Cutler (ALLHAT [9,10] [Antihypertensive and Lipid-Lowering Treatment to Prevent Heart Attack Trial]),
B R Davis (ALLHAT [9,10]),
R B Devereaux (LIFE [37,38] [Losartan Intervention For Endpoint reduction in hypertension]),
J Dwyer (IDNT [60] [Irbesartan Diabetic Nephropathy Trial]),
R Estacio (ABCD [5–7] [Appropriate Blood Pressure Control in Diabetes]),
R Fagard (Syst-Eur [50] [SYSTolic Hypertension in EURope]),
K Fox (EUROPA [28,29] [European trial on Reduction Of cardiac events with Perindopril among patients with stable coronary Artery disease]),
T Fukui (CASE-J [17,18] [Candesartan Antihypertensive Survival Evaluation in Japan]),
A K Gupta (ASCOT-BPLA [12,13] [Anglo-Scandinavian Cardiac Outcomes Trial – Blood Pressure Lowering Arm]),
R R Holman (UKPDS [54]),
Y Imai (HOMED-BP [33] [Hypertension Objective Treatment Based on Measurement by Electrical Devices of Blood Pressure]),
M Ishii (JMIC-B [36] [Japan Multicenter Investigation for Cardiovascular Diseases-B]),
S Julius (VALUE [52,53]),
Y Kanno (E-COST [61] [Efficacy of Candesartan on Outcome in Saitama Trial]),
S E Kjeldsen (VALUE, [52,53] LIFE [37,38]),
J Kostis (SHEP [62] [Systolic Hypertension in the Elderly Program]),
K Kuramoto (NICS-EH [40] [National Intervention Cooperative Study in Elderly Hypertensives]),
J Lanke (STOP Hypertension-2 [49] [Swedish Trial in Old Patients with Hypertension-2], NORDIL [63] [Nordic Diltiazem]),
E Lewis (IDNT [60]),
J Lewis (IDNT [60]),
M Lievre (DIABHYCAR [24,25] [Non-insulin-dependent diabetes, hypertension, microalbuminuria or proteinuria, cardiovascular events, and ramipril study]),
L H Lindholm (CAPPP [49] [Captopril Prevention Project], STOP Hypertension-2,[49] NORDIL [63]),
S Lueders (MOSES [39] [The Morbidity and Mortality After Stroke, Eprosartan Compared With Nitrendipine for Secondary Prevention]),
S MacMahon (ADVANCE [58]),
M Matsuzaki (COPE [23] [The Combination Therapy of Hypertension to Prevent Cardiovascular Events]),
M H Mehlum (VALUE [52,53]),
S Nissen (CAMELOT [16] [Comparison of Amlodipine vs Enalapril to Limit Occurrences of Thrombosis]),
H Ogawa (HIJ-CREATE [32] [Heart Institute of Japan Candesartan Randomized Trial for Evaluation in Coronary Heart Disease]),
T Ogihara (CASE-J,[17,18] COLM [19,20] [Combinations of OLMesartan], COPE [23])
T Ohkubo (HOMED-BP [33]),
C Palmer (INSIGHT [57]),
A Patel (ADVANCE [58]),
C J Pepine (INVEST [35] [International Verapamil SR-Trandolapril Study]),
M Pfeffer (PEACE [64] [Prevention of Events with Angiotensin- Converting Enzyme Inhibition]),
N R Poulter (ASCOT-BPLA [12,13] [Anglo-Scandinavian Cardiac Outcomes Trial]),
H Rakugi (CASE-J,[17,18] VALISH [65] [Valsartan in Elderly Isolated Systolic Hypertension Study]),
G Reboldi (Cardio-Sis [66] [CARDIOvascolari del Controllo della Pressione Arteriosa SIStolica]),
C Reid (ANBP2 [11] [The Second Australian National Blood Pressure Study]),
G Remuzzi (BENEDICT [14,15] [BErgamo NEphrologic DIabetes Complications Trial]),
P Ruggenenti (BENEDICT [14,15]),
T Saruta (CASE-J [17,18]),
J Schrader (MOSES [39]),

R Schrier (deceased) (ABCD [5–7]),
P Sever (ASCOT-BPLA [12,13]),
P Sleight (deceased) (CONVINCE,[21,22] HOPE [34] [Heart Outcomes Prevention Evaluation], ONTARGET [41,42] [Ongoing Telmisartan Alone and in Combination with Ramipril Global Endpoint Trial], TRANSCEND [41,51] [Telmisartan Randomised AssessmeNt Study in ACE iNtolerant subjects with cardiovascular Disease]),
J A Staessen (Syst-Eur [50])
H Suzuki (E-COST [61]),
L Thijs (Syst-Eur [50]),
K Ueshima (CASE-J,[17,18] VALISH [65]),
S Umemoto (COPE [23]),
W H van Gilst (PREVEND IT [44]),
P Verdecchia (Cardio-Sis [66] [CARDIOvascolari del Controllo della Pressione Arteriosa SIStolica]),
K Wachtell (LIFE [37,38]),
L Wing (ANBP2 [11]),
M Woodward (ADVANCE,[58] PROGRESS [59]),
Y Yui (JMIC-B [36]),
S Yusuf (HOPE,[34] ONTARGET,[41,42] PRoFESS,[47,48] TRANSCEND [41,51]),
A Zanchetti (deceased) (ELSA [26,27] [European Lacidipine Study on Atherosclerosis], VHAS [67] [Verapamil in Hypertension and Atherosclerosis Study])
Z Y Zhang (Syst-Eur [50])

Other members: C Anderson, C Baigent, BM Brenner, R Collins, D de Zeeuw, J Lubsen, E Malacco, B Neal, V Perkovic, B Pitt, A Rodgers, P Rothwell, G Salimi-Khorshidi, J Sundström, F Turnbull, G Viberti, J Wang.

Steering Committee

Kazem Rahimi (Chair) (Nuffield Department of Women's and Reproductive Health, University of Oxford, Oxford, UK), Koon K Teo (Population Health Research Institute, McMaster University, Hamilton, Ontario, Canada) Barry R Davis (The University of Texas School of Public Health, Houston, Texas, USA), John Chalmers (The George Institute for Global Health, University of New South Wales, Sydney, Australia), Carl J Pepine (Department of Medicine, University of Florida, Gainesville, Florida, USA).

References

1 Rahimi K, Canoy D, Nazarzadeh M, *et al.* Investigating the stratified efficacy and safety of pharmacological blood pressure-lowering: an overall protocol for individual patient-level data meta-analyses of over 300 000 randomised participants in the new phase of the Blood Pressure Lowering Treatm. *BMJ Open* 2019; **9**: e028698.

2 Austin PC, Lee DS, Fine JP. Introduction to the Analysis of Survival Data in the Presence of Competing Risks. *Circulation* 2016; **133**: 601–9.

3 Wright JT, Bakris G, Greene T, *et al.* Effect of blood pressure lowering and antihypertensive drug class on progression of hypertensive kidney disease: Results from the AASK trial. *J Am Med Assoc* 2002; **288**: 2421–31.

4 Gassman JJ, Greene T, Wright JT, *et al.* Design and statistical aspects of the African American Study of Kidney Disease and Hypertension (AASK). *J Am Soc Nephrol* 2003; **14**: 154–65.

5 Estacio RO, Schrier RW. Antihypertensive therapy in type 2 diabetes: Implications of the Appropriate Blood Pressure Control in Diabetes (ABCD) trial. *Am J Cardiol* 1998; **82**: 9–14.

6 Estacio RO, Savage S, Nagel NJ, Schrier RW. Baseline characteristics of participants in the appropriate blood pressure control in diabetes trial. *Control Clin Trials* 1996; **17**: 242–57.

7 Estacio RO, Jeffers BW, Hiatt WR, Biggerstaff SL, Gifford N SR. The effect of nisoldipine as compared with enalapril on cardiovascular outcomes in patients with non-insulin-dependent diabetes and hypertension. *N Engl J Med* 1998; **338**: 645–52.

8 The ACTIVE I Investigators, Yusuf S, Healey JS, *et al.* Irbesartan in patients with atrial fibrillation. *N Engl J Med* 2011; **364**: 928–38.

9 Davis BR, Cutler JA, Gordon DJ, *et al.* Rationale and design for the antihypertensive and lipid lowering treatment to prevent heart attack trial (ALLHAT). *Am J Hypertens* 1996; **9**: 342–60.

10 ALLHAT Officers and Coordinators for the ALLHAT Collaborative Research Group. Major Outcomes in High-Risk Hypertensive Patients Randomized to Angiotensin-Converting Enzyme Inhibitor or Calcium Channel Blocker vs Diuretic. *JAMA J Am Med Assoc* 2002; **288**: 2981–97.

11 Second Australian National Blood Pressure Study Group. A comparison of outcomes with angiotensin-converting--enzyme inhibitors and diuretics for hypertension in the elderly. *N Engl J Med* 2015; **348**: 687–96.

12 Sever PS, Dahlöf B, Poulter NR, *et al.* Rationale, design, methods and baseline demography of participants of the Anglo-Scandinavian cardiac outcomes trial. *J Hypertens* 2001; **19**: 1139–47.

13 Dahlöf B, Sever PS, Poulter NR, *et al.* Prevention of cardiovascular events with an antihypertensive regimen of amlodipine adding perindopril as required versus atenolol adding bendroflumethiazide as required, in the Anglo-Scandinavian Cardiac Outcomes Trial-Blood Pressure Lowering Arm (ASCOT-B. *Lancet* 2005; **366**: 895–906.

14 BENEDICT Group. The BErgamo NEphrologic DIabetes Complications Trial (BENEDICT): Design and baseline characteristics. *Control Clin Trials* 2003; **24**: 442–61.

15 Ruggenenti P, Fassi A, Ilieva AP, *et al.* Preventing microalbuminuria in type 2 diabetes. *N Engl J Med* 2004; **351**: 1941–51.

16 Nissen SE, Tuzcu EM, Libby P, *et al.* Effect of antihypertensive agents on cardiovascular events in patients with coronary disease and normal blood pressure: the CAMELOT study: a randomized controlled trial. *JAMA J Am Med Assoc* 2004; **292**: 2217–25.

17 Ogihara T, Nakao K, Fukui T, *et al.* Effects of candesartan compared with amlodipine in hypertensive patients with high cardiovascular risks: Candesartan antihypertensive survival evaluation in Japan trial. *Hypertension* 2008; **51**: 393–8.

18 Fukui T, Rhaman M, Hayashi K, *et al.* Candesartan Antihypertensive Survival Evaluation in Japan (CASE-J) trial of cardiovascular events in high-risk hypertensive patients: Rationale, design, and methods. *Hypertens Res* 2003; **26**: 979–90.

19 Ogihara T, Saruta T, Rakugi H, *et al.* Rationale, study design and implementation of the COLM study: The combination of OLMesartan and calcium channel blocker or diuretic in high-risk elderly hypertensive patients. *Hypertens Res* 2009; **32**: 163–7.

20 Ogihara T, Saruta T, Rakugi H, *et al.* Combinations of olmesartan and a calciumchannel blocker or a diuretic inelderly hypertensive patients: A randomized, controlled trial. *J Hypertens* 2014; **32**: 2054–63.

21 Black HR, Elliott WJ, Neaton JD, *et al.* Rationale and design for the controlled ONset Verapamil INvestigation of cardiovascular endpoints (CONVINCE) trial. *Control Clin Trials* 1998; **19**: 370–90.

22 Black HR, Elliott WJ, Grandits G, *et al.* Principal Results of the Controlled Onset Verapamil Investigation of Cardiovascular End Points (CONVINCE) Trial. *J Am Med Assoc* 2003; **289**: 2073–82.

23 Matsuzaki M, Ogihara T, Umemoto S, *et al.* Prevention of cardiovascular events with calcium channel blocker-based combination therapies in patients with hypertension: A randomized controlled trial. *J Hypertens* 2011; **29**: 1649–59.

24 Marre M, Lievre M, Chatellier G, Mann JFE, Passa P, Ménard J. Effects of low dose ramipril on cardiovascular and renal outcomes in patients with type 2 diabetes and raised excretion of urinary albumin: Randomised, double blind, placebo controlled trial (the DIABHYCAR study). *Br Med J* 2004; **328**: 495–9.

25 DIABHYCAR Study Group. The non-insulin-dependent diabetes, hypertension, microalbuminuria or

proteinuria, cardiovascular events, and ramipril (DIABHYCAR) study: Design, organization, and patient recruitment. *Control Clin Trials* 2000; **21**: 383–96.
26 Bond G, Dal Palú C, Hansson L, *et al.* The E.L.S.A. trial: protocol of a randomized trial to explore the differential effect of antihypertensive drugs on atherosclerosis in hypertension. *J Cardiovasc Pharmacol* 1994; **23**: S85-87.
27 Zanchetti A, Bond MG, Hennig M, *et al.* Calcium antagonist lacidipine slows down progression of asymptomatic carotid atherosclerosis: Principal results of the European Lacidipine Study on Atherosclerosis (ELSA), a randomized, double-blind, long-term trial. *Circulation* 2002; **106**: 2422–7.
28 Gomma AH, Fox KM, EUROPA Investigators. The EUROPA trial: Design, baseline demography and status of the substudies. *Cardiovasc Drugs Ther* 2001; **15**: 169–79.
29 Fox KM, Bertrand M, Ferrari R, *et al.* Efficacy of perindopril in reduction of cardiovascular events among patients with stable coronary artery disease: Randomised, double-blind, placebo-controlled, multicentre trial (the EUROPA study). *Lancet* 2003; **362**: 782–8.
30 The European Working Party on High Blood Pressure in the Elderly (EWPHE): An international trial of antihypertensive therapy in elderly patients. Objectives, protocol and organization. *Arch Int Pharmacodyn Ther* 1985; **275**: 300–34.
31 Amery A, Brixko P, Clement D, *et al.* Mortality and morbidity results from the European Working Party on High Blood Pressure in the Elderly trial. *Lancet* 1985; **1**: 1349–54.
32 Kasanuki H, Hagiwara N, Hosoda S, *et al.* Angiotensin II receptor blocker-based vs. non-angiotensin II receptor blocker-based therapy in patients with angiographically documented coronary artery disease and hypertension: The Heart Institute of Japan Candesartan Randomized Trial for Evaluation in . *Eur Heart J* 2009; **30**: 1203–12.
33 Asayama K, Ohkubo T, Metoki H, *et al.* Cardiovascular outcomes in the first trial of antihypertensive therapy guided by self-measured home blood pressure. *Hypertens Res* 2012; **35**: 1102–10.
34 The Heart Outcomes Prevention Evaluation Study Investigators. Effect of ramipril on cardiovascular events in high-risk patients. *N Engl J Med* 2000; **342**: 145–53.
35 Pepine C, Handberg EM, Cooper-dehoff RM, *et al.* A calcium antagonist vs a non-calcium antagonist hypertension treatment strategy for patients with coronary artery disease. The International Verapamil-Trandolapril Study (INVEST): a randomized controlled trial. 2003; **290**.
36 Yui Y, Sumiyoshi T, Kodama K, *et al.* Comparison of nifedipine retard with angiotensin converting enzyme inhibitors in Japanese hypertensive patients with coronary artery disease: The Japan Multicenter Investigation for Cardiovascular Diseases-B (JMIC-B) randomized trial. *Hypertens Res* 2004; **27**: 181–91.
37 Dahlöf B, Devereux RB, Julius S, *et al.* Characteristics of 9194 patients with left ventricular hypertrophy: The LIFE study. *Hypertension* 1998; **32**: 989–97.
38 Dahlöf B, Devereux RB, Kjeldsen SE, *et al.* Cardiovascular morbidity and mortality in patients with diabetes in the Losartan Intervention For Endpoint reduction in hypertension study (LIFE): A randomised trial against atenolol. *Lancet* 2002; **359**: 1004–10.
39 Schrader J, Lüders S, Kulschewski A, *et al.* Morbidity and mortality after stroke, eprosartan compared with nitrendipine for secondary prevention: Principal results of a prospective randomized controlled study (MOSES). *Stroke* 2005; **36**: 1218–24.
40 National Intervention Cooperative Study in Elderly Hypertensives Study Group. Randomized double-blind comparison of a calcium antagonist and a diuretic in elderly hypertensives: National intervention cooperative study in elderly hypertensives study group. *Hypertension* 1999; **34**: 1129–33.
41 The ONTARGET/TRANSCEND Investigators. Rationale, design, and baseline characteristics of 2 large, simple, randomized trials evaluating telmisartan, ramipril, and their combination in high-risk patients: The Ongoing Telmisartan Alone and in Combination with Ramipril Global Endpoint Trial/Telmi. *Am Heart J* 2004; **148**: 52–61.
42 The ONTARGET Investigators. Telmisartan, ramipril, or both in patients at high risk for vascular events. *N Engl J Med* 2008; **358**: 1547–59.
43 MacMahon S, Sharpe N, Gamble G, *et al.* Randomized, placebo-controlled trial of the angiotensin-converting enzyme inhibitor, ramipril, in patients with coronary or other occlusive arterial disease. *J Am Coll Cardiol* 2000; **36**: 438–43.
44 Asselbergs FW, Diercks GFH, Hillege HL, *et al.* Effects of fosinopril and pravastatin on cardiovascular events in subjects with microalbuminuria. *Circulation* 2004; **110**: 2809–16.
45 Byington RP, Miller ME, Herrington D, *et al.* Rationale, design, and baseline characteristics of the Prospective Randomized Evaluation of the Vascular Effects of Norvasc Trial (PREVENT). *Am J Cardiol* 1997; **80**: 1087–90.
46 Pitt B, Byington RP, Furberg CD, *et al.* Effect of amlodipine on the progression of atherosclerosis and the occurrence of clinical events. *Circulation* 2000; **102**: 1503–10.
47 Yusuf S, Diener HC, Sacco RL, *et al.* Telmisartan to prevent recurrent stroke and cardiovascular events. *N Engl J Med* 2008; **359**: 1225–37.
48 Diener HC, Sacco R, Yusuf S. Rationale, design and baseline data of a randomized, double-blind, controlled trial comparing two antithrombotic regimens (a fixed-dose combination of extended-release dipyridamole plus ASA with clopidogrel) and telmisartan versus placebo in patients with. *Cerebrovasc Dis* 2007; **23**: 368–80.

49 Hansson L, Lindholm LH, Ekbom T, *et al.* Randomised trial of old and new antihypertensive drugs in elderly patients: Cardiovascular mortality and morbidity the Swedish trial in old patients with hypertension-2 study. *Lancet* 1999; **354**: 1751–6.

50 Staessen JA, Fagard R, Thijs L, *et al.* Randomised double-blind comparison of placebo and active treatment for older patients with isolated systolic hypertension. The Systolic Hypertension in Europe (Syst-Eur) Trial Investigators. *Lancet* 1997; **350**: 757–64.

51 The TRANSCEND Investigators. Effects of the angiotensin-receptor blocker telmisartan on cardiovascular events in high-risk patients intolerant to angiotensin-converting enzyme inhibitors: a randomised controlled trial. *Lancet* 2008; **372**: 1174–83.

52 Mann J, Julius S. The Valsartan Antihypertensive Long-term Use Evaluation (VALUE) trial of cardiovascular events in hypertension. Rationale and design. *Blood Press* 1998; **7**: 176–83.

53 Julius S, Kjeldsen SE, Weber M, *et al.* Outcomes in hypertensive patients at high cardiovascular risk treated with regimens based on valsartan or amlodipine: The VALUE randomised trial. *Lancet* 2004; **363**: 2022–31.

54 UK Prospective Diabetes Study Group. Tight blood pressure control and risk of macrovascular and microvascular complications in type 2 diabetes: UKPDS 38. *Br Med J* 1998; **317**: 703–13.

55 The Dutch TIA Trial Study Group. Trial of secondary prevention with atenolol after transient ischemic attack or nondisabling ischemic stroke. *Stroke* 1993; **24**: 543–8.

56 HYVET Study Group. Treatment of Hypertension in Patients 80 Years of Age or Older. *N Engl J Med* 2008; **358**: 1887–98.

57 Brown MJ, Palmer CR, Castaigne A, *et al.* Morbidity and mortality in patients randomised to double-blind treatment with a long-acting calcium-channel blocker or diuretic in the International Nifedipine GITS study: Intervention as a Goal in Hypertension Treatment (INSIGHT). *Lancet* 2000; **356**: 366–72.

58 ADVANCE Collaborative Group. Effects of a fixed combination of perindopril and indapamide on macrovascular and microvascular outcomes in patients with type 2 diabetes mellitus (the ADVANCE trial): a randomised controlled trial. *Lancet* 2007; **370**: 829–40.

59 PROGRESS Collaborative Group. Randomised trial of a perindopril-based blood-pressure-lowering regimen among 6105 individuals with previous stroke or transient ischaemic attack. *Lancet* 2001; **358**: 1033–41.

60 The Collaborative Study Group. Renoprotective effect of the angiotensin-receptor antagonist irbesartan in patients with nephropathy due to type 2 diabetes. *N Engl J Med* 2011; **345**: 851–60.

61 Suzuki H, Kanno Y, Kanai A, *et al.* Effects of candesartan on cardiovascular outcomes in Japanese hypertensive patients. *Hypertens Res* 2005; **28**: 307–14.

62 SHEP Cooperative Research Group. Prevention of stroke by antihypertensive drug treatment in older persons with isolated systolic hypertension. Final results of the Systolic Hypertension in the Elderly Program (SHEP). *JAMA J Am Med Assoc* 1991; **265**: 3255–64.

63 Hedner T. The nordic diltiazem study (NORDIL). A prospective intervention trial of of calcium antagonist therapy in hypertension. *Blood Press* 1993; **2**: 312–21.

64 The PEACE Trial Investigators. Angiotensin-Converting–Enzyme Inhibition in Stable Coronary Artery Disease. *N Engl J Med* 2004; **351**: 2058–68.

65 Ogihara T, Saruta T, Matsuoka H, *et al.* Valsartan in Elderly Isolated Systolic Hypertension (VALISH) study: Rationale and design. *Hypertens Res* 2004; **27**: 657–61.

66 Verdecchia P, Staessen JA, Angeli F, *et al.* Usual versus tight control of systolic blood pressure in non-diabetic patients with hypertension (Cardio-Sis): an open-label randomised trial. *Lancet* 2009; **374**: 525–33.

67 Rosei EA, Dal Palù C, Leonetti G, Magnani B, Pessina A, Zanchetti A. Clinical results of the verapamil in hypertension and atherosclerosis study. *J Hypertens* 1997; **15**: 1337–44.

BPLTTC Research Protocol

Project: Cancer in BPLTTC
Date: 28 Aug 2019
Version: 082019_05

Protocol title:
Effects of antihypertensive use on the risk of cancer, stratified by drug class

Contributors (to date):
Emma Copland
Dexter Canoy
Milad Nazarzadeh
Mark Woodward
Kazem Rahimi
On behalf of the Blood Pressure Lowering Treatment Trialists' Collaboration

Background

Hypertension is one of the most prevalent chronic conditions, affecting over one billion people globally (1). The prevalence and incidence of hypertension is increasing further due to population ageing and growth, as well as an increase in the prevalence of cardiometabolic risk factors, including high BMI and lack of physical activity. Hypertension is an important risk factor for cardiovascular disease, therefore, the World Health Organisation has pledged to reduce the prevalence of hypertension by 25% between 2010 and 2025. Antihypertensive medication is an important tool for controlling blood pressure, and although less than one in five hypertensive individuals have their blood pressure controlled, millions of individuals are prescribed antihypertensive drugs globally. While the evidence for the benefits of antihypertensive medication in the reduction of cardiovascular disease is well-established (2), there have been some concerns about possible unintended consequences in the use of these drugs, including increasing the risk of developing cancer (3–6). In the absence of any evidence for an association between blood pressure and cancer risk (7), it seems unlikely that blood pressure reduction per se increases the risk of cancer. However, several hypotheses have been posited linking the pathways of specific drug classes to cancer, independently of a change in blood pressure. For example, the blockade of angiotensin II receptors by angiotensin II receptor blockers (ARBs) has been implicated in increased cell proliferation, angiogenesis and tumour progression (8). Additionally, several antihypertensive drugs across different classes, particularly thiazide diuretics, have photosensitising properties that could increase susceptibility to skin cancer (9). Nevertheless, the evidence for an increased risk of cancer overall or by specific type with the use of different antihypertensive classes has been inconsistent and even conflicting. Meta-analyses of observational studies have suggested that ARBs are not associated with skin (5), prostate (10) or breast cancer (11). Conflicting meta-analyses of observational studies have been reported, with some suggesting that thiazide diuretics are associated with an increased risk of skin cancer (4,5) but others suggesting there is no association (6). Calcium channel blockers (CCBs) and beta-blockers have also been linked to an increased risk of skin cancer (6) and there is some evidence that angiotensin-converting-enzyme inhibitors (ACEis) have a protective effect on breast (11) and skin cancer (5). However, observational studies suffer from inherent biases and residual confounding. Evidence from a meta-analysis of nine randomised controlled trials (RCTs) suggested that participants using ARBs had an increased risk of cancer compared to control groups (3), but two subsequent meta-analyses of RCTs did not find any association between ARBs and cancer risk (12,13). While one meta-analysis of RCTs did not find a link between any drug class and the incidence of cancer overall (13), it could not rule out an increased risk of cancer overall with the use of ACEis in combination with ARBs; this meta-analysis also did not investigate specifically the association between thiazide diuretics and skin cancer risk. Findings from existing trials and their meta-analyses are currently limited as it is possible that such investigations lacked statistical power to investigate cancer incidence due to small numbers of events.

The third cycle of the Blood Pressure Lowering Treatment Trialists' Collaboration (BPLTTC) provides the largest individual patient-level data (IPD) on blood pressure-lowering trials currently available. Cancer outcomes were requested from all trials when invited to join the collaboration, providing a large number of cancer events that were not published and therefore not available for most aggregate patient-data level meta-analyses. The availability of IPD also offers an opportunity to investigate the effect of antihypertensive drug classes on cancer risk across a number of important patient groups.

Objectives

In this proposed IPD meta-analysis, we aim to investigate the effect of use of specific antihypertensive drug classes on the risk of cancer. More specifically, we aim to:

1) Investigate the effect of each antihypertensive drug class (ACEis, ARBs, beta-blockers, CCBs and diuretics) on the risk of any cancer and cancer mortality
2) Investigate the effect of specific antihypertensive drug classes on specific cancer types (colon, lung, breast, prostate, skin cancers), particularly where associations have been identified in the literature, depending on power:
    a. ARBs and all common cancer types
    b. Diuretics and skin cancers
    c. CCBs and skin cancers
    d. Beta-blockers and skin cancers
    e. ACEis and breast and skin cancers
3) Investigate the effect of antihypertensive drug classes on the risk of developing cancer in patient subgroups, defined by age at baseline and sex-specific analyses for breast and prostate cancer, as well as other cancer types depending on statistical power

Methods

Study design
This study is an IPD meta-analysis of blood pressure-lowering trials which examines the effects of antihypertensive drug classes on the incidence of cancer.

Eligibility criteria
All trials that met criteria described previously and have contributed IPD to the collaboration will be considered (14). The search criteria was extended to include the period between 1st June 2018 and 1st September 2019. Eligible trials are those that have provided data on cancer outcomes and have a study design that compares at least one drug class (or drug class combination) with a control group (Appendix table).

Study population
All participants from the eligible trials will be included in the analysis.

Interventions
In this meta-analysis, the intervention is pharmacologic lowering of blood pressure using various antihypertensive drug classes. Both placebo-controlled trials and trials of drug class (or drug class combination) comparisons are included.

Outcome variables
The primary outcome is any cancer event, defined as the first cancer event diagnosed after randomisation in participants. These cancer events include those pre-specified as outcomes as well as those reported as adverse events in each trial. Some trials reported both non-fatal and fatal cancer events while others only reported cancer mortality. Cancer events were reported using ICD codes, MedDRA classifications, by cancer grouping or as any cancer or cancer death. The primary outcome comprises first cancer event recorded through at least one of these methods. The secondary outcomes comprise cancer mortality and cancer incidence by major cancer grouping (lung, prostate, breast, colon, skin, other). The type of outcome data provided by each trial will be described (illustrative table 2).

Comparison groups
Analyses will be conducted for comparisons between each antihypertensive drug class (ACEis, ARBs, beta-blockers, CCBs and diuretics) and all other comparator groups, including placebo, standard treatments or other drug classes (or drug class combinations). We will also conduct separate analyses on placebo-controlled trials for drug classes where we have sufficient data.

In the stratified analyses according to patient subgroups, we will categorise patients according to:
1. Age (<60, 60 to 69, 70 to 79 and ≥80 years)
2. Sex (men and women)
3. Use of blood pressure-lowering treatment at baseline (yes vs no) [this could be either a pre-specified subgroup or part of sensitivity analyses – might depend on numbers]
4. Smoking status (current smokers and past or never smokers)
5. BMI (normal weight: 18.5 to 25kg/m^2 and overweight/obese: >25kg/m^2)

Age categories may be combined to ensure that there are sufficient numbers of patients within each of the category.

Sample size considerations
The exact numbers of cancer events in each comparison group are not known at this stage, therefore we estimate the number of cancer events required to achieve 80% power for a range of effect sizes based on published cancer data for individual trials included in the analysis (Table 1). The R package 'powerSurvEpi' was used for the sample size estimations.

The proportions of participants with cancer events in the two largest trials for ACEi *vs* other (ALLHAT and ONTARGET) range from 7.7% to 9.1% (13). If the number of events in our dataset is within this range we expect to have enough events to detect a HR of 1.07 with 80% power.

The two largest trials in the ARB *vs* other group (ONTARGET and VALUE) report the overall proportion of participants with cancer events as 7.2% and 9.1% (13). Assuming that the proportion of participants in our dataset with cancer events is 9% overall, we expect to have 80% power to detect a HR as low as 1.065. Two other trials (LIFE and TRANSCEND) report proportions of 7.3% and 7.4% (13). If the proportion of participants with cancer events in our dataset overall is 7% we expect to have enough events to detect a HR of 1.08 with 80% power in the ARB *vs* other group.

The largest trial in the BB *vs* other comparison group (ASCOT-BPLA) reports that 9.8% of participants developed cancer during the trial (13). Assuming that the proportion of participants with cancer events in our dataset is 9.8%, we expect to have 80% power to detect a HR of 1.10. Another trial (LIFE) reported the overall the proportion of participants with

cancer events as 7.3% (13). If the proportion of participants with cancer events in our dataset overall is 7.3% we expect to have enough events to detect a HR of 1.15 with 80% power.

The proportions of participants with cancer events in the largest trials for CCB *vs* other (ALLHAT, ASCOT-BPLA and VALUE) range from 7.2% to 9.8% (13). If the number of events in our dataset is at the lower end of this range we expect to have enough events to detect a HR of 1.06 with 80% power, at the higher end we expect to have enough events to detect a HR of 1.05 with 80% power. Two other large trials (CONVINCE and INVEST) report that between 1.7% and 3.7% of participants developed cancer (13). Assuming that the proportion of participants with cancer events in our dataset is 2%, we expect to have 80% power to detect a HR of 1.15.

The only trial with published cancer data included in the diuretic *vs* comparison group (ALLHAT) reports that 7.7% of participants developed cancer during follow-up (13). Assuming that the proportion of participants with cancer events in our dataset is 7.7%, we expect to have 80% power to detect a HR of 1.09.

Assessment of trials
The trials included in the analysis will be assessed for risk of bias using the Cochrane risk-of-bias tool (15).

Statistical analysis
The IPD meta-analyses will be conducted using the one-stage approach (16,17). The baseline characteristics of the participants in each drug class comparison group will be described (illustrative table 3). For continuous variables mean values and standard deviations will be reported; for categorical variables, the frequencies and proportions will be reported. The analyses will be based on intention-to-treat principle – we will included all eligible participants regardless of whether they received the allocated treatment. Time-to-event analyses will be conducted using clustered Cox proportional hazards models. These mixed effects models will include a random effects term for trial to take into account clustering at the trial level. The start time of the analysis is defined as the date of randomisation for each participant. Individuals are censored at their last follow-up date or the date of a competing risk event such as death that is not cancer-related. Methods that account for informative censoring will be used to take into account the presence of competing risks (18).

The effects of antihypertensive drugs on total cancer events will also be investigated in different patient subgroups. The pre-specified subgroups will be defined by age, sex, baseline use of antihypertensive drugs. Table 4 is an illustrative table to summarise the effects of antihypertensive use on the risk of cancer by drug class. Heterogeneity at the trial-level or across subgroups will be assessed through χ^2 tests, including χ^2 tests for trend where appropriate. Results for all analyses will be presented using forest plots.

Sensitivity analysis
A number of sensitivity analyses will be undertaken. We will compare results separately for trials that reported adjudicated cancer events to those that recorded unadjudicated cancer events to determine whether the difference in adjudication of events impacts the results of the analysis. In the main analysis, trials reporting both non-fatal and fatal cancer events and those only reporting cancer mortality will be pooled together to maximise statistical power. In a sensitivity analysis, we will perform separate analyses on these two types of trials and compare the results. We will also conduct a sensitivity analysis where any individuals with pre-existing cancer at baseline will be excluded.

The main analyses will be limited to trials for which we have IPD; however, excluding trials where IPD is not available could introduce bias. To investigate this possibility, published results or aggregate data from eligible trials for which we do not have IPD will be combined with the IPD available in a sensitivity analysis. We will extract hazard ratios (HRs) and 95% confidence intervals (CIs) from published papers where available, and tabular data will be extracted from trials where HR estimates are not reported. IPD, published HR estimates (and 95% CIs) and tabular data will be pooled in a meta-analysis using random effects. This method will account for information on censoring where available. Relative risks (RRs) will be reported for these analyses. Through this approach, the consistency of the findings with and without inclusion of eligible trials for which we do not have IPD will be evaluated. Meta-regression will be used if considerable residual heterogeneity remains after controlling for all possible variables and recognized effect modifiers, with P-value adjusted for false positive findings based on Monte Carlo simulation. Funnel plots could also be generated to assess potential selection bias associated with inclusion of trials based on availability of IPD.

We will investigate the application of network meta-analysis models to our analysis. These models allow the synthesis of individual trials with different treatment comparisons, thereby combining both direct and indirect evidence of relative treatment effects (19). This method may be useful where simple pairwise comparisons between drug classes are not possible due to small numbers of trials or where there is no direct comparison. Recently, methods for conducting network meta-analysis using individual patient-level information have been developed, although the adoption of this method remains limited (20,21). If assessed to be informative, we will use this method to indirectly estimate relative treatment effects from RCTs with different treatment comparisons. Novel network meta-analysis methods incorporate both IPD and Bayesian/mixed effects frameworks (22,23).

For the main analyses we will report summary HRs with their 95% CIs with P values tested at 5% significance level (two-tailed). To account for the increased possibility of obtaining a chance finding due to multiple testing in the subgroup analyses, tests will be conducted at the more stringent significance level of 1% (two-tailed) and estimates will be reported with 99% CIs (24).

Table 1. Number of events needed for an 80% power to detect a statistically significant difference in risk over a range of risk estimates. HR = hazard ratio.

Drug class comparison	N of trials	Treatment: control ratio	Number of events needed overall to detect HR with 80% power							
			1.05	***1.06***	***1.07***	***1.08***	***1.09***	***1.10***	***1.15***	***1.20***
ACEi *vs* other	13	0.47	14,715	10,285	7605	5860	4660	3795	1740	1010
ARB *vs* other	10	0.76	13,190	9235	6845	5285	4210	3440	1595	935
BB *vs* other	4	0.93	13,025	9130	6770	5235	4175	3415	1590	935
CCB *vs* other	18	0.63	13,585	9505	7040	5430	4325	3530	1630	950
Diuretic *vs* other	6	0.63	13,585	9505	7040	5430	4325	3530	1630	950

TABLES AND FIGURES FOR ILLUSTRATIVE PURPOSES ONLY

Table 2. Definition and quality of cancer outcomes in each trial. Type of cancer outcome recorded in each trial (any cancer, cancer deaths, first cancer whether or not it is fatal, cancer subtype), details on whether the cancer events where adjudicated (pre-specified outcomes) or not (adverse events) and whether the date of diagnosis was provided. [*Illustrative purpose only*.]

**Trial**	**Type of cancer outcome reported in trial**	**Adjudicated outcome**	**Date of event provided**
AASK			
ABCD			
ACTIVE I			
ALLHAT			
ANBP2			
ASCOT-BPLA			
BENEDICT			
CAMELOT			
CASE-J			
COLM			
CONVINCE			
COPE			
DIABHYCAR			
ELSA			
EUROPA			
EWPHE			
HIJ-CREATE			
HOMED-BP			
HOPE			
INVEST			
JMIC-B			
LIFE			
MOSES			
NICS-EH			
ONTARGET			
PART-2			
PREVEND IT			
PREVENT			
PROFESS			
STOP-Hypertension-2			
Syst-Eur			
TRANSCEND			
VALUE			

Table 3. Summary details of trials included in the BPLTTC individual patient-level data meta-analysis. [*Illustrative purpose only*.]

**Characteristics**	**ACEi vs other**	**ARB vs other**	**BB vs other**	**CCB vs other**	**Diuretic vs other**
N of trials					
N of participants (% women)					
% Caucasian/European ethnicity (N)					
% current smoker (N)					
Mean (SD) pre-treatment SBP/DBP					
Mean (SD) achieved SBP/DBP					
Mean (SD) age (years)					
N of participants by age (years) at baseline					
<50					
50 to 59					
60 to 69					
70 to 79					
≥80					
Mean (SD) trial duration (years)					
% with condition at baseline (N)					
Cardiovascular disease					
Diabetes					
Chronic kidney disease					
% previously on blood pressure-lowering medication (N)					
No. of participants by year of end of trial (N of trials)					
<1990					
1990 to 1999					
2000 to 2009					
≥2010					
N of participants/trials with alcohol intake data					
Mean (SD) alcohol intake (g/day)					
N of participants/trials with BMI data					
Mean (SD) BMI (kg/m^2)					

Table 4. Effects of antihypertensive use on overall cancer incidence by drug class, overall and in pre-specified patient subgroups. [*Illustrative purpose only*.]

	Hazard ratio (95% confidence interval) for cancer incidence				
	ACEi vs other	ARB vs other	BB vs other	CCB vs other	Diuretic vs other
**All**					
**By age (years)**					
<60					
60-69					
70-79					
≥80					
**By sex**					
Women					
Men					
**By baseline BP medication**					
Yes					
No					
**By smoking status**					
Current smoker					
Ex- or never smoker					
**Body mass index**					
18.5 to 25kg/m^2 (normal weight)					
>25kg/m^2 (overweight/obese)					

Appendix Table. Summary of blood pressure-lowering treatment randomised trials included in the analysis.

Study name or author	Publication year	Drug class comparisons	N
**AASK** (African American Study of Kidney Disease and Hypertension)	2002	ACEi *vs* CCB *vs* BB and more vs less intense BP-lowering	1094
**ABCD** (Appropriate Blood Pressure Control in Diabetes Trial)	1998	CCB *vs* ACEi	950
**ACTIVE I** (Atrial Fibrillation Clopidogrel Trial with Irbesartan for Prevention of Vascular Events)	2011	ARB *vs* placebo	9016
**ALLHAT** (Antihypertensive and Lipid Lowering Treatment to Prevent Heart Attacks Trial)	2002	ACEi *vs* CCB *vs* Diuretic	42,418
**ANBP2** (Second Australian National Blood Pressure Study)	2003	ACEi *vs* Diuretic	6083
**ASCOT-BPLA** (Anglo-Scandinavian Cardiac Outcomes Trial - Blood Pressure Lowering Arm)	2005	CCB(+ACEi) *vs* BB(+Diuretic)	19,257
**BENEDICT** (Bergamo Nephrologic Diabetes Complications Trial)	2004	ACEi *vs* CCB *vs* ACEi+CCB *vs* placebo	1209
**CAMELOT** (The Comparison of Amlodipine vs Enalapril to Limit Occurrences of Thrombosis)	2004	ACEi *vs* CCB *vs* placebo	1997
**CASE-J** (Candesartan Antihypertensive Survival Evaluation in Japan)	2008	ARB *vs* CCB	4703
**COLM** (Combination of OLMesartan and calcium channel blocker or diuretic)	2014	CCB+ARB *vs* Diuretic+ARB	5141
**CONVINCE** (Controlled Onset Verapamil Investigation of Cardiovascular End Points)	2003	CCB *vs* BB/Diuretic	16,476
**COPE** (Combination Therapy of Hypertension to Prevent Cardiovascular Events)	2011	ARB+CCB *vs* BB+CCB *vs* Diuretic+CCB	3293
**DIABHYCAR** (Noninsulin-dependent diabetes, hypertension, microalbuminuria or proteinuria, cardiovascular events, and ramipril)	2004	ACEi *vs* placebo	4912
**ELSA** (European Lacidipine Study on Atherosclerosis)	2002	CCB *vs* BB	2334
**EUROPA** (European trial on reduction of cardiac events with perindopril in stable coronary artery)	2003	ACEi *vs* placebo	12,218
**EWPHE** (European Working Party on High Blood Pressure in the Elderly)	1985	Diuretic *vs* placebo	840
**HIJ-CREATE** (Heart Institute of Japan Candesartan Randomized Trial for Evaluation in Coronary Heart Disease)	2009	ARB *vs* non-ARB	2049
**HOMED-BP** (Hypertension Objective Treatment based on Measurement by Electrical Devices of Blood Pressure Study)	2012	ACEi *vs* ARB *vs* CCB	3518
**HOPE** (Heart Outcomes Prevention Evaluation Study)	2000	ACEi *vs* placebo	9297
**INVEST** (International Verapamil-Trandolapril Study)	2003	CCB *vs* non-CCB	22,576
**JMIC-B** (Japan Multicenter Investigation for Cardiovascular Diseases-B)	2004	CCB *vs* ACEi	1650
**LIFE** (Losartan Intervention for Endpoint Reduction in Hypertension Study)	2002	ARB *vs* BB	9193
**MOSES** (Morbidity and Mortality After Stroke, Eprosartan Compared With Nitrendipine for Secondary Prevention)	2005	ARB *vs* CCB	1352
**NICS-EH** (National Intervention Cooperative Study in Elderly Hypertensives)	1999	CCB *vs* Diuretic	429
**ONTARGET** (Ongoing Telmisartan Alone and in Combination with Ramipril Global Endpoint Trial)	2008	ACEi *vs* ARB *vs* ACEi+ARB	25,620
**PART-2** (Prevention of Atherosclerosis with Ramipril Trial)	2000	ACEi *vs* placebo	617
**PREVEND IT** (Prevention of Renal and Vascular Endstage Disease)	2004	ACEi *vs* placebo	864
**PREVENT** (Prospective Randomized Evaluation of the Vascular Effects of Norvasc Trial)	2000	CCB *vs* placebo	825
**PROFESS** (Prevention Regimen for Effectively Avoiding Second Strokes)	2003	ARB *vs* placebo	19,798
**STOP Hypertension-2** (Swedish Trial in Old Patients with Hypertension-2)	1999	ACEi *vs* CCB *vs* BB+Diuretic	6614
**Syst-Eur** (Systolic Hypertension in Europe)	1997	CCB *vs* placebo	4695
**TRANSCEND** (Telmisartan Randomised Assessment Study in ACE Intolerant Subjects with Cardiovascular Disease)	2008	ARB *vs* placebo	5926
**VALUE** (Valsartan Antihypertensive Long-Term Use Evaluation)	2004	ARB *vs* CCB	15,245
**Total participants**			**260,447**

References

1. Yusuf S, Wood D, Ralston J, Reddy KS. The World Heart Federation's vision for worldwide cardiovascular disease prevention. Lancet. 2015; 386(9991):399–402.
2. Blood Pressure Lowering Treatment Trialists' Collaboration. Effects of different blood-pressure-lowering regimens on major cardiovascular events: results of prospectively-designed overviews of randomised trials. Lancet. 2003; 362(9395):1527–35.
3. Sipahi I, Debanne SM, Rowland DY, Simon DI, Fang JC. Angiotensin-receptor blockade and risk of cancer: meta-analysis of randomised controlled trials. Lancet Oncol. 2010; 11(7):627–36. A
4. Shin D, Lee ES, Kim J, Guerra L, Naik D, Prida X. Association Between the Use of Thiazide Diuretics and the Risk of Skin Cancers: A Meta-Analysis of Observational Studies. J Clin Med Res. 2019
5. Tang H, Fu S, Zhai S, Song Y, Asgari MM, Han J. Use of antihypertensive drugs and risk of keratinocyte carcinoma: A meta-analysis of observational studies. Pharmacoepidemiol Drug Saf. 2018; 27(3):279–88.
6. Gandini S, Palli D, Spadola G, Bendinelli B, Cocorocchio E, Stanganelli I, et al. Anti-hypertensive drugs and skin cancer risk: a review of the literature and meta-analysis. Crit Rev Oncol Hematol. 2018; 122:1–9.
7. Lacey B, Lewington S, Clarke R, Kong XL, Chen Y, Guo Y, et al. Age-specific association between blood pressure and vascular and non-vascular chronic diseases in 0·5 million adults in China: a prospective cohort study. Lancet Glob Heal. 2018; 6(6):e641–9.
8. Link WT, De Felice A. An FDA overview of rodent carcinogenicity studies of angiotensin II AT-1 receptor blockers: Pulmonary adenomas and carcinomas. Regul Toxicol Pharmacol. 2014; 70(2):555–63.
9. Kreutz R, Algharably EAH, Douros A. Reviewing the effects of thiazide and thiazide-like diuretics as photosensitizing drugs on the risk of skin cancer. J Hypertens. 2019 ; 1.
10. Cao L, Zhang S, Jia C, He W, Wu L, Li Y, et al. Antihypertensive drugs use and the risk of prostate cancer: a meta-analysis of 21 observational studies. BMC Urol. 2018; 18(1):17.
11. Ni H, Rui Q, Zhu X, Yu Z, Gao R, Liu H. Antihypertensive drug use and breast cancer risk: a meta-analysis of observational studies. Oncotarget. 2017; 8(37):62545–60.
12. ARB Trialists Collaboration. Effects of telmisartan, irbesartan, valsartan, candesartan, and losartan on cancers in 15 trials enrolling 138 769 individuals. J Hypertens. 2011; 29(4):623–35.
13. Bangalore S, Kumar S, Kjeldsen SE, Makani H, Grossman E, Wetterslev J, et al. Antihypertensive drugs and risk of cancer: Network meta-analyses and trial sequential analyses of 324 168 participants from randomised trials. Lancet Oncol. 2011; 12(1):65–82.
14. Rahimi K, Canoy D, Nazarzadeh M, Salimi-Khorshidi G, Woodward M, Teo K, et al. Investigating the stratified efficacy and safety of pharmacological blood pressure-lowering: an overall protocol for individual patient-level data meta-analyses of over 300 000 randomised participants in the new phase of the Blood Pressure Lowering Treatm. BMJ Open. 2019; 9(5):e028698.
15. Sterne J, Savović J, Page M, Elbers R, Blencowe N, Boutron I, et al. RoB 2: a revised tool for assessing risk of bias in randomised trials. BMJ. 2019;
16. Debray TPA, Moons KGM, van Valkenhoef G, Efthimiou O, Hummel N, Groenwold RHH, et al. Get real in individual participant data (IPD) meta-analysis: a review of the methodology. Res Synth Methods. 2015; 6(4):293–309.
17. Legha A, Riley RD, Ensor J, Snell KIE, Morris TP, Burke DL. Individual participant data meta-analysis of continuous outcomes: A comparison of approaches for specifying and estimating one-stage models. Stat Med. 2018; 37(29):4404–20.
18. Austin PC, Lee DS, Fine JP. Introduction to the Analysis of Survival Data in the Presence of Competing Risks. Circulation. 2016; 133(6):601–9.
19. Caldwell DM, Ades AE, Higgins JPT. Simultaneous comparison of multiple treatments: combining direct and indirect evidence. BMJ. 2005; 331(7521):897–900.
20. Woods B, Hawkins N, Mealing S, Sutton A, Abraham WT, Beshai JF, et al. Individual patient data network meta-analysis of mortality effects of implantable cardiac devices. Heart. 2015; 101(22):1800–6.
21. Ribassin-Majed L, Marguet S, Lee AWM, Ng WT, Ma J, Chan ATC, et al. What Is the Best Treatment of Locally Advanced Nasopharyngeal Carcinoma? An Individual Patient Data Network Meta-Analysis. J Clin Oncol. 2017; 35(5):498.
22. Debray TP, Schuit E, Efthimiou O, Reitsma JB, Ioannidis JP, Salanti G, et al. An overview of methods for network meta-analysis using individual participant data: when do benefits arise? Stat Methods Med Res. 2018; 27(5):1351–64.
23. Freeman SC, Carpenter JR. Bayesian one-step IPD network meta-analysis of time-to-event data using Royston-Parmar models. Res Synth Methods. 2017; 8(4):451–64.
24. Sjölander A, Vansteelandt S. Frequentist versus Bayesian approaches to multiple testing. Eur J Epidemiol. 2019; 1–13.
